# Recently Deglaciated High-Altitude Soils of the Himalaya: Diverse Environments, Heterogenous Bacterial Communities and Long-Range Dust Inputs from the Upper Troposphere

**DOI:** 10.1371/journal.pone.0076440

**Published:** 2013-09-26

**Authors:** Blaz Stres, Woo Jun Sul, Bostjan Murovec, James M. Tiedje

**Affiliations:** 1 Department of Animal Science, University of Ljubljana, Domzale, Slovenia; 2 Center for Microbial Ecology, Michigan State University, East Lansing, Michigan, United States of America; 3 Department of Systems Biotechnology, Chung-Ang University, Anseong, Korea; 4 Faculty of Electrical Engineering, University of Ljubljana, Ljubljana, Slovenia; U. S. Salinity Lab, United States of America

## Abstract

**Background:**

The Himalaya with its altitude and geographical position forms a barrier to atmospheric transport, which produces much aqueous-particle monsoon precipitation and makes it the largest continuous ice-covered area outside polar regions. There is a paucity of data on high-altitude microbial communities, their native environments and responses to environmental-spatial variables relative to seasonal and deglaciation events.

**Methodology/Principal Findings:**

Soils were sampled along altitude transects from 5000 m to 6000 m to determine environmental, spatial and seasonal factors structuring bacterial communities characterized by 16 S rRNA gene deep sequencing. Dust traps and fresh-snow samples were used to assess dust abundance and viability, community structure and abundance of dust associated microbial communities. Significantly different habitats among the altitude-transect samples corresponded to both phylogenetically distant and closely-related communities at distances as short as 50 m showing high community spatial divergence. High within-group variability that was related to an order of magnitude higher dust deposition obscured seasonal and temporal rearrangements in microbial communities. Although dust particle and associated cell deposition rates were highly correlated, seasonal dust communities of bacteria were distinct and differed significantly from recipient soil communities. Analysis of closest relatives to dust OTUs, HYSPLIT back-calculation of airmass trajectories and small dust particle size (4–12 µm) suggested that the deposited dust and microbes came from distant continental, lacustrine and marine sources, e.g. Sahara, India, Caspian Sea and Tibetan plateau. *Cyanobacteria* represented less than 0.5% of microbial communities suggesting that the microbial communities benefitted from (co)deposited carbon which was reflected in the psychrotolerant nature of dust-particle associated bacteria.

**Conclusions/Significance:**

The spatial, environmental and temporal complexity of the high-altitude soils of the Himalaya generates ongoing disturbance and colonization events that subject heterogeneous microniches to stochastic colonization by far away dust associated microbes and result in the observed spatially divergent bacterial communities.

## Introduction

Himalaya (from Sanskrit *him* = snow, *alaya* = abode) refers to the complex system of nearly parallel ranges of tertiary mountains, stretching over 3000 km, from Myanmar on its east and Afghanistan on its west. While the Himalayan system rises abruptly from the plains of India, it stretches to the north in series of folds to the High Plateau of Tibet, forming a complex series of several nearly parallel or converging and bifurcating ranges, intersected by high longitudinal valleys and plateaus. The width of the system is extremely variable as it spans from only 80 km to more than 300 km giving rise to a unique distribution of elevation (4–8.8 km), slope (30°–90°), temperature gradients and fluctuations (−25°C/+15°C), low partial pressure (at 6000 m only 46% of that at sea level), winds (40–160 km h^−1^) and UV irradiation (10 kJ m^−2^ day^−1^). Its altitude and close proximity to the highly energetic tropical environment [1]; [2] form a barrier to atmospheric transport, which produces much of the region’s aqueous and particle monsoon precipitation [3] and makes the Himalaya the largest continuous ice-covered area outside polar regions.

This region is characterized by strong winds from the SW in the (summer) monsoon season and from NW in the dry (winter) season, thunderstorms accompanied by heavy precipitation in the pre-monsoon season and at the onset of winter [4]. The mean annual precipitation as snow was 2500–3000 mm [5]; [6]. The sky is cloudy 10% and 90% of winter and summer monsoons, respectively [7]. The dust concentrations at 5000 m can range from 8–100 µg m^−3^ up to 800 µg m^−3^ and are highest in spring [8] providing an annual dust flux to the Himalayan range spanning from 770 to 1030 mg m^−2^ [9]. The dust plume over the Tibetan plateau to the north of the sampling area covers more than half of the Tibetan plateau in the 6–8 km altitude. Only dust particles smaller than 20 µm diameter can be lifted into the high atmosphere in spring by sufficiently strong storms [10]. The dust north-south transport is blocked by the Himalayas generating high temporal and spatial deposition variability [11] due to high monsoon and orographically forced precipitation, air-mass turbulence and surface ruggedness [12]; [13]; [5]; [14].

The recent loss of snow cover and thawing of the permafrost is changing the conditions for microorganisms in these regions. Photographs collected during expeditions taken during the last 25 years in the Kanchenjunga region show that the snow cover has been significantly receding at least since 1988. Satellite image analysis [15]; [16] and field explorations [17] show a 3% increase in barren or alpine grass covered area per decade at the expense of snow-covered area, with the soils now characterized as non-continuous permafrost. The landscape is further shaped by water run-off and daily freeze-thaw [18].

Despite this, abundant bacteria were detected in these 5000 m to 6000 m altitude soils at up to 10^8^ cell g^−1^ by microscopic enumeration and qPCR, some were viable on various media (CFU or MPN) with counts from 2˙10^4 ^g^−1^ to 3˙10^6 ^g^−1^ at 4°C and up to 2˙10^7 ^g^−1^ at 28°C (unpublished), and they exhibited versatile and freeze-thaw resistant metabolism as measured from 4°C respiration of different substrates [18]. However, little is known about the interplay of environmental characteristics and bacterial microbial community structure at elevations several thousand meters above sea level. We hypothesized that the 1000 m difference in altitude and habitat types would result in qualitative differences in microbial community structure as assessed by 454 pyrosequencing of 16 S rRNA genes and would enable identification of the key structuring environmental parameters. Secondly, based on the observation that permanent snow cover at 6000 m disappeared by 2005 we hypothesized that microbial communities at the now barren sites would differ significantly from previously glaciated communities and exhibit rearrangements due to seasonal shifts in environmental conditions. Last, dust and snow-ice samples collected in 2002, 2005, 2006 and 2007 were used to quantify the aeolian sedimentation in order to identify and quantify the incoming dust-associated bacteria and assess their viability. Underestimation of the size of the global dust cycle was reported recently implying that the deposition flux of dust and its fertilizing effects on ecosystems were substantially larger than thought before [19]. Hence, given the long-range dust influx and the harsh resident conditions [18], our third hypothesis was that the dust associated microbial communities were significantly different from the receiving soil microbial communities.

## Materials and Methods

### Ethics Statement

Field studies carried out on high-altitude mostly barren deglaciated sites did not involve endangered or protected species. No specific permissions were required other than on-site supervision of Liaison Officer of Government of Nepal.

### Sample Site and Sampling Design

The study was conducted on shallow, high-altitude, non-continuous permafrost soils of a south-facing, alpine ridge descending from Drohmo peak (6980 m), Nepal (27° 48′ 00″ N and 88° 07′ 02″ E; Figure S1 in File S1) [18]. Surface soil samples were collected in October 2002 along 200 m horizontal transects at each 200 m vertical increment starting from 5000 m to 6000 m (Figure S2 in File S1), resulting in 24 sampled locations (Table S1 in File S1). At the 6000 m location, transect samples were additionally collected in October 2005 and April 2006.

The samples were stored at 4°C or less in a glacier crevasse, airlifted and kept cold until arrival in the laboratory. All soil physical-chemical attributes previously described for microcosm experiments were determined as described before [18]. Patchiness was defined as a difference in elevation between the sampling and surrounding area (d = 2 m) greater than 0.4 m. Surface characteristics were analyzed using Arc-GIS at resolution of 25 m. For spatial interpolation of environmental gradients with altitude and transect width the spherical, exponential, Gaussian and cubic models were tested. Semivariograms were constructed using the best (cubic) model, cross validated by jackknifing after which kriging algorithm was used [20].

### Snow and Dust Trap Samples

The extent of bacterial and dust particle deposition from the atmosphere was tested during October 2002, October 2005 and April 2006 (Table S1 in File S1). Dust traps were installed 15 cm above the highest point next to each soil sampling site and left in place for 30 days (four tubes per transect) in 2002. Sterile 50 mL Falcon tubes were aseptically filled with a 2 cm layer of 100% polyester wool that was pretreated with a solution of 10% formaldehyde in 70% glycerol to prevent *in-situ* microbial growth. One replicate per altitude was lost or displaced due to fierce winds leaving three replicates per each 200 m altitude transect. After collection the tubes were capped, transported to the lab and rinsed with sterile 0.22 um filtered bidistilled water. The rinse water was collected on a membrane filter (0.22 µm). Particle diameter measurements, microbial cell staining and counting were performed as described before [21]; [18]. The number of particles and microorganisms was expressed per unit area (per square centimeter of Falcon tube surface area (2.9 cm diameter)) per exposure time (30 days). To follow dust deposition dynamics at 6000 m additional dust traps were set up in 2005 and 2006 (Table S1 in File S1).

Snow samples were collected aseptically at a separate nearby location (6012 m; 27° 47′ 55″N, 88°07′ 32″E) in 2005, 2006 and 2007 (Table S1 in File S1) and thus represent a short-term subset of the 30-day dust trap experiment. Seven sterile 50 mL Falcon tubes were used to collect pristine surface snow by immersing the tube mouth into snow immediately after snowfall at several random locations within 3 m^2^, allowed to thaw and fixed with formaldehyde (final concentration of 3.9%) to prevent microbial growth. The volume of thawed snow was determined gravimetrically and fixed samples were analyzed as described above.

Three replicates of 6000 m snow sampled in autumn 2005, spring 2006 and the autumn 2007 were not preserved by formaldehyde. One mL aliquots from each tube were plated on 100× diluted nutrient broth solid medium. The fraction of inoculated plates exhibiting microbial growth was recorded after six week incubation at 4°C.

Due to expected low biomass in snow and dust samples precautions were taken to exclude possible contamination of samples. Sterile Falcon tubes were used by experimenters. Single use vinyl gloves were used on sampling sites. When Falcon tube containers were put in place or used for sampling, the corresponding screw caps were replaced with those taken from spare sterile Falcon tubes to close after exposure. For snow sampling empty tubes were manipulated in the same way as those used for sampling snow except that no snow was collected and served as controls for contamination. Tubes were opened, exposed to air for the same period of time as during snow sampling and processed as described above.

### DNA Extraction, PCR and DNA Sequencing

DNA was extracted from triplicate representative 0.5 g samples for each of the 24 transect points and the additional 6000 m samples with a MoBio UltraClean Soil DNA extraction kit. Dust samples were aseptically filtered as described for particle and cell enumeration in a grade A laminar hood. Filters were aseptically cut into small pieces, loaded into the bead tube of a DNA extraction kit and the resulting DNA from dust associated microorganisms extracted as done for soil. The background control for dust samples consisted of identical treatment of unexposed dust collection tubes followed by direct enumeration and PCR. DNA concentration and purity were determined spectrophotometrically (Nanodrop 1000, Thermo Scientific). The three replicate dust samples collected in 2005 and 2006 were pooled to achieve sufficient DNA for sequencing (Table S1 in File S1). One soil sample from transect 5000 m had insufficient DNA, leaving 23 samples for spatial analyses. DNA of dust and soil was amplified by the previously described primer set 2 (mixed reverse primers) under the described conditions [22]. Amplicons were sequenced using the Genome Sequencer FLX System (454 Life Science, Bradford, CT, USA) at the Michigan State University Research Technology Support Facility.

### Sequence Analysis

Raw pyrosequencing reads were sorted by barcode, quality-filtered (reads shorter than 150 bps or with more than 2 mismatches in both primer regions were removed) by the RDP pyrosequencing pipeline [23]. Remaining sequences were clustered into operational taxonomic units (OTUs) at 97% identity using complete-linkage clustering [23]. Taxonomic assignment was by the RDP Classifier using a 70% confidence threshold [24].

All sequences were combined into a single file, dereplicated and aligned in Mothur [25] and Good’s coverage determined [25] (Table S2 in File S1). Intra- and inter-sample comparisons were calculated using Jest, Jabund, Bray-Curtis and ThetaYC values as implemented in Mothur. The significance of differences between samples was assessed by pairwise parsimony and Unifrac tests. Phylogenetic trees were constructed using the approximately maximum-likelihood algorithm in Fasttree V2.1.3, GTR evolutionary model, 30 rounds of minimum evolution-nearest neighbour interchange, followed by 15 rounds of maximum likelihood nearest neighbour interchange [26].

To explore the patterns in source locations of OTU representatives, the closely related sequences were obtained from RDP II using the Seqmatch utility in GenBank format (KNN matches = 10). The resulting database of closely related sequences was analyzed for entries in GenBank file line ‘isolation source = ’ using custom bioperl script.

Sequence data and metadata obtained in this study were deposited in MG-RAST metagenomics analysis server [27] under MG-RAST ID: 4528669.3.

### Data Analysis

Soil physical-chemical parameters and habitat characteristics were first analyzed for significant differences between two or more groups using non-parametric MANOVA (NP-MANOVA) [28] based on Bray-Curtis distance in PAST [29]. SIMPER was used to identify the environmental factors responsible for an observed difference between groups of samples and the overall significance of the differences was assessed by one-way ANOSIM.

Differences in environmental characteristics and OTU composition were visualized using non-metric multidimensional scaling (NM-MDS) based on OTU Bray-Curtis distance [30]. Convex hulls, i.e. the smallest convex polygon enclosing all the points of a particular sample in environmental and community structure analyses were used as a designation of occupied trait space [30]; [31].

The relative spatial distances between sampling points were derived from field measurements and GPS coordinates using C++ script. For the visualization of OTU level distance-decay relationships, the Bray-Curtis pairwise dissimilarities were calculated for all 23 spatial samples based on arc sin square root transformed species relative abundances.

The variation partitioning between environmental variables, spatial coordinates of the samples and patterns in bacterial community structure were examined by multivariate analyses using the software package CANOCO V4.5 [32] as described before [18]; [33]; [34].

To compute airmass back trajectories for the duration of all dust sampling experiments we used HYSPLIT_4 (Hybrid Single Particle Lagrangian Integrated Trajectory; http://ready.arl.noaa.gov/HYSPLIT.php) Model accessed via OAA Air Resources Laboratory, Silver Spring, USA (http://ready.arl.noaa.gov) [35]. Average monthly aerosol amounts were based on the Moderate Resolution Imaging Spectroradiometer (MODIS: http://modis.gsfc.nasa.gov/) on NASA’s Terra satellite (http://terra.nasa.gov/) obtained from the NASA’s Earth Observatory (http://earthobservatory.nasa.gov/).

## Results and Discussion

### Environmental Gradients

**Figure 1:**
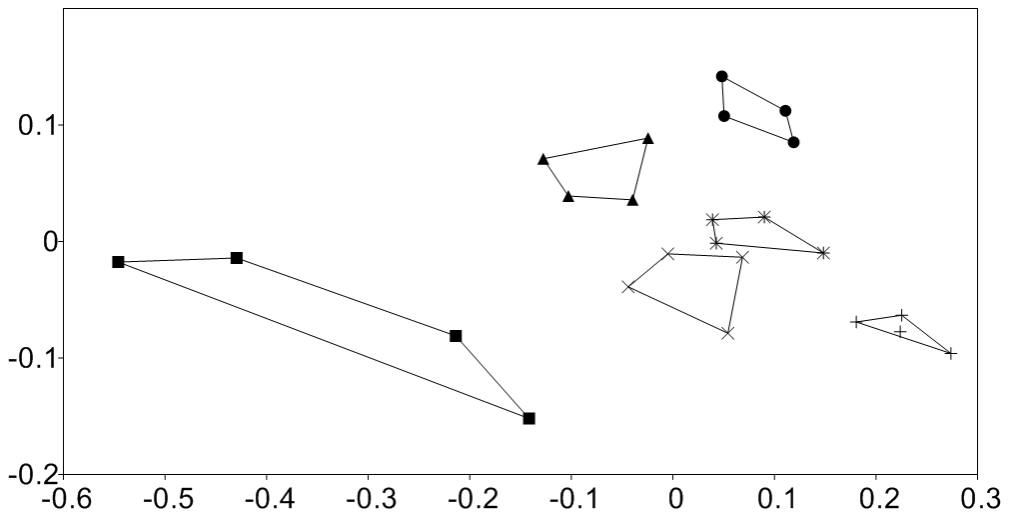
Non-metric multidimensional scaling ordination of 5000–6000 m altitudinal gradient soil characteristics. (•) 6000 m, (▴) 5800 m, (×) 5600 m, (_*_) 5400 m, (+) 5200 m, (▪) 5000 m. Individual samples connected by lines define convex hull surface area of NM-MDS scores of environmental characteristics as a measure of trait-space covered.

Significant differences in physical-chemical parameters existed among altitude groups of samples (NP-MANOVA, p<0.05): C/N ratio, slope, nitrate and coarse sand were identified by SIMPER as environmental factors primarily responsible for the observed differences between altitude groups of samples, contributing 93.1% of the total group difference of 2002 soil samples (Figure 1). Monte-Carlo (MC) permutation test was used to assess the significance of correlations, final stress (0.11), instability (0.001) and the number of iterations in NM-MDS (173) indicated a stable 2 D solution (P<0.01). The proportions of variance represented by each axis, based on the r^2^ between distance in the ordination space and distance in the original *n-*dimensional space were 0.79 and 0.15, respectively. The 6000 m samples from 2002, 2005 and 2006 differed significantly in soil moisture content only.

The sites exhibited a narrow range of soil particle size classes but varied widely with respect to moisture, water holding capacity and carbon content, slope angle and habitat types (Figure S3 in File S1). Strong Pearson’s correlations were observed between (i) clay vs. C/N ratio or reducing sugars (r>0.85); (ii) reducing sugars vs. slope or altitude (r>0.72); (iii) C and N vs. habitat type, plant community or WHC (r>0.67). Other parameters measured were not well correlated with one another (−0.5<r<0.5).

Except for the 5200 m moraine and 5400 m slope, all other sites exhibited significant patchiness, as differences in the surface configuration exceeded 0.5 m due to uneven surface topology, gravel piles, boulders and water runoff streams (Figure S5 in File S1). The 5200 m nearly flat area, and the 5400 m and 5600 m slopes were covered in grass, grass-moss and moss communities similar to polar tundra, and characterized by the sorted patterned ground microtopology [36]: the 5200 m site exhibited regularly shaped frost cracks, ice wedge polygons as observed before on nearly horizontal polar soil surface [37]; [36]. Frost stripes and irregular polygons were observed at much steeper 5400 m and 5600 m sites whereas the barren 5000 m, 5800 m and 6000 m sites surfaces were dominated by deglaciated gravel, pebbles and boulders, respectively.

Convex hull surface area of NM-MDS scores of environmental characteristics was highest in barren 5000 m glacier surface site. Lowest values were recorded for the vegetated 5200 m site (Figure 1). An increase in convex hull area as a measure of trait-space was observed for vegetated sites from 5200 m to 5600 m with a subsequent decrease in 5800 m and 6000 m barren sites (Figure 1) suggesting a varying degree of high spatial heterogeneity in environmental characteristics. Indeed, complex gradients in environmental parameters with altitude or transect length were detected (Figure S4 in File S1) contrary to previous reports on glacier forefield soils in the Andes or Antarctica [37]; [38]; [39] showing that the Himalayan sites differed significantly from those reported before in slope steepness, altitude and surface ruggedness.

### General Analyses of the Pyrosequencing Datasets

A total of 89,567 (average 2800 sample^−1^) V4 sequences were obtained for 31 Himalayan soil and dust samples, which resulted in 7,800 OTUs. No single OTU accounted for >0.11% of the entire dataset. A comparison of unique sequences in each sample revealed remarkably little overlap in OTUs as up to 1,521 OTUs were observed in a single sample, whereas no common (core) OTUs could be determined in 23 or all 31 spatio-temporal samples.

The sequences were classified to 27 phyla: *Acidobacteria, Proteobacteria, Verrucomicrobia, Actinobacteria, Gemmatimonadetes, Planctomycetes, Firmicutes, Bacteroidetes, Cyanobacteria,* TM7, *Deinococcus-Thermus, Nitrospira,* OD1, Bacteria_incertae_sedis (Class *Ktedonobacteria*), *Fusobacteria, Chlamydiae,* OP10, WS3, BRC1, *Chloroflexi, Lentisphaerae, Spirochaetes,* SR1, *Synergistetes, Tenericutes and Thermotogae*. In contrast, temperate soils with 6 to 8.5 times more sequences revealed only 17 and 23 phyla and 2–3 times fewer OTUs [40], [41]. Further, *Ktedonobacteria, Fusobacteria, Lentispherae, SR1, Synergistetes, Tenericutes* and *Thermotogae*, many of them ubiquitous in diverse environments, were detected in the Himalayan soils in addition to 19 out of 20 phyla detected in Antarctic glacier foreland [38]. The four generally most abundant bacterial phyla: *Acidobacteria, Proteobacteria, Verrrucomicrobia* and *Actinobacteria* were encountered at an average frequency 21% (5–41%), 19.8% (8–45%), 14.9% (4–39%), 10.3% (1–27%) (min–max of each library’s relative abundance), respectively. The relative taxa distribution between the sampling sites in the Himalayan altitude transects was highly variable although the dominant taxa present at the landscape scale corresponded with those reported for other temperate soils. NM-MDS and NP-MANOVA analyses (Figure S6 in File S1) revealed that the composition of the Himalayan microbial communities was significantly different from other temperate soils, volcanic sediments, permafrost, polar and Antarctic soils, marine and fresh-water sediments (p<0.0001; n = 149 samples; [22]).

### Spatial Distribution of Bacterial Communities and Impact of Soil Properties

**Figure 2:**
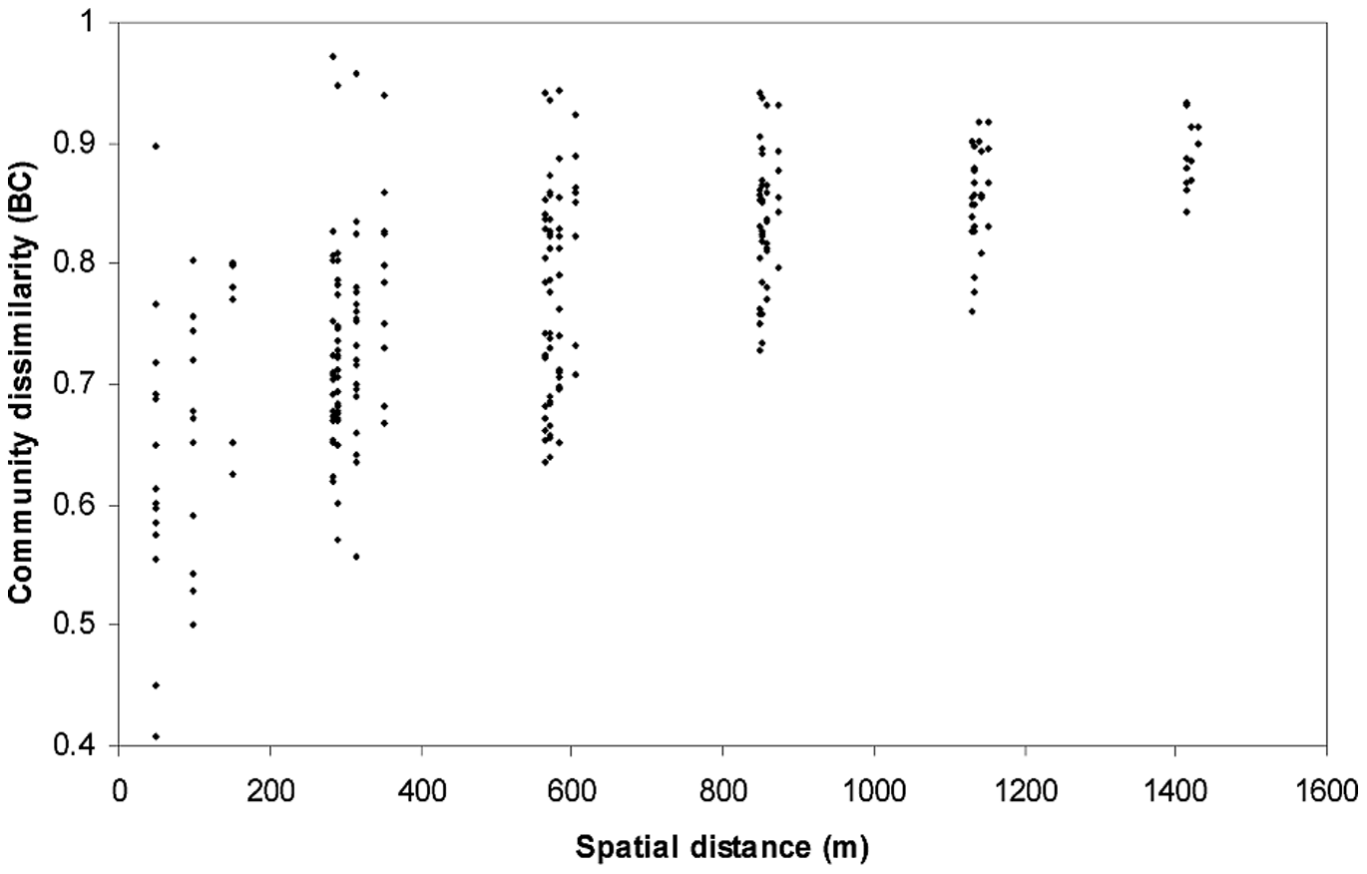
Distance-decay relationships in the non-continuous permafrost soils of the 1000 m altitude gradient. Pairwise dissimilarities (Bray-Curtis index) of microbial communities were plotted as a function of the distance (vertical plus horizontal) between the sampling locations.

Significant landscape scale spatial pattern of microbial community distribution was identified (Figure 2) up to a distance of more than 1,400 m (vertical plus horizontal distances). Spatial categories were highly separated in NP-MANOVA (p<0.001). The community dissimilarity increased with distance, but the span of dissimilarities in closely located communities (<200 m) was high (0.41<d<0.91) in comparison to that of distantly (>1400 m) located communities that were generally more dissimilar (0.85<d<0.97). In contrast, at distances shorter than 240 m at Niwot Ridge [20], a much lower elevation alpine site in Colorado (3250 m), more closely related communities were reported while it was equally likely to find closely or distantly related communities at distances larger than 240 m. In the Himalaya, distantly related communities were likely at distances larger than 800 m, while it was equally likely to find closely or distantly related communities at distances as short as 50 m.

**Figure 3:**
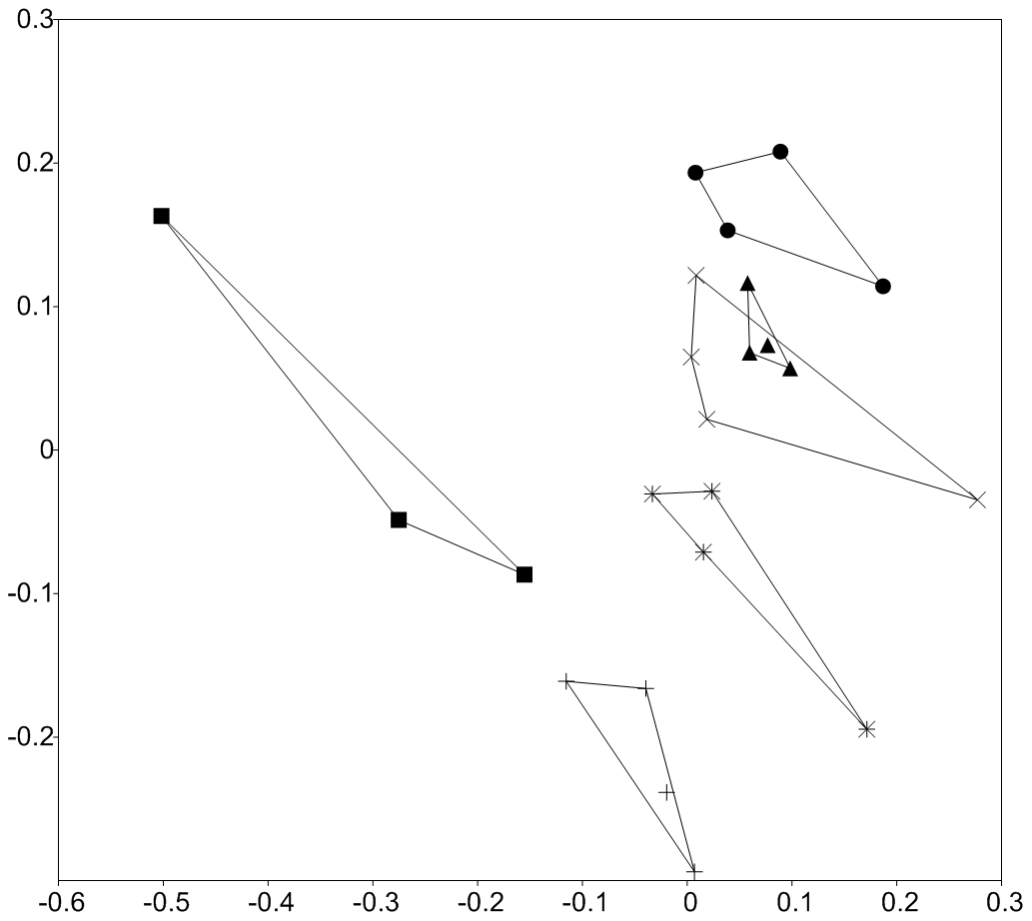
Non-metric multidimensional scaling ordination of microbial communities sampled from 5000–6000 m altitudinal gradient. (•) 6000 m, (▴) 5800 m, (×) 5600 m, (_*_) 5400 m, (+) 5200 m, (▪) 5000 m. Horizontal transect formed significant groups (NP-MANOVA, p<0.05). Filled symbols denote barren soils. Individual samples connected by lines define convex hull surface area of NM-MDS scores of microbial community characteristics as a measure of community-space covered.

**Figure 4:**
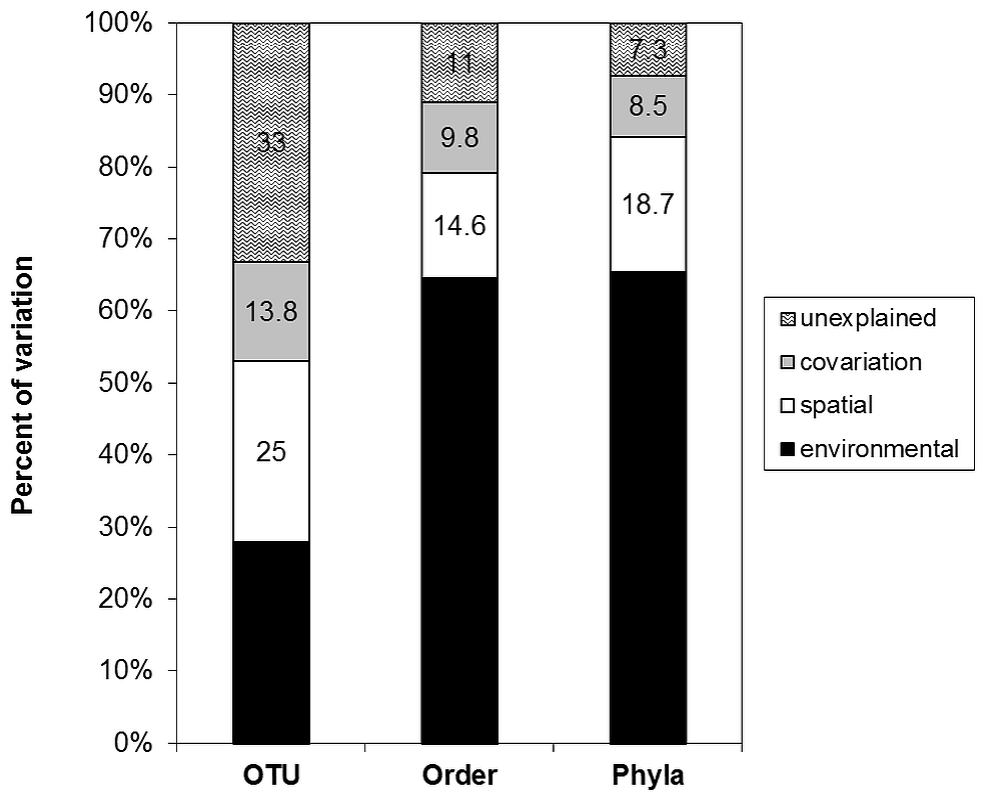
Variance partitioning of environmental and spatial factors shaping microbial communities at the three phylogenetic levels (OTU, Order, Phylum).

Pairwise parsimony and Unifrac tests identified significant spatial differences (p<0.05) in community structure between (i) samples from the different locations and (ii) samples from the same year and altitude (i.e. within transect). Between altitude transect groups of microbial communities (i.e. between transects) also differed significantly (NP-MANOVA; p<0.05)(Figure 3). Stress decomposition (final stress 4.32), final instability (<10^−4^), the number of iterations (n = 84) (P<0.004) indicated a stable two dimensional ordination. The proportion of variance represented by each axis based on the r^2^ between distance in the ordination space and distance in the original *n-*dimensional space were 0.45 and 0.39. The microbial community convex hull area (Figure 3) as a measure of community trait-space did not reflect environmental trait-space characteristics (Figure 1) suggesting low correspondence between the two datasets. Previous Antarctic and Alpine studies of microbial communities showed that spatial, temporal and seasonal patterns in community structure, microbial diversity and metabolic activities could be explained by environmental parameters or the general community history [37]; [38]; [42] without considering the covariability of environmental characteristics with space. In this study, environmental and spatial characteristics explained a comparable fraction of variability (28% and 25%) at the level of OTU, respectively (Figure 4). Covariability of the two explained an additional 13.8% leaving 33.2% of variability unexplained, inline with the complex spatial distribution of measured environmental characteristics (Figure S4 in File S1). Although 66.8% of community structure variability could be explained at the level of OTU, significantly associated environmental parameters (Table 1) contributed only 28% of explained variability when corrected for spatial correlation. The low proportion of covariability suggests that environmental parameters do not scale well with space, confirming high spatial heterogeneity of the site. The distinct environmental parameters that significantly explained community structure (Table 1) largely corresponded to those identified in previous soil studies. In contrast, surface patchiness (Figure S5 in File S1) was identified (Table 1) to be significantly associated with bacterial community structure across all three phylogenetic levels, which has not been noted before in studies analyzing temperate or glacier forefield soils [38]; [39]; [40].

**Table 1:** Conditional effects of environmental variables selected by forward selection in redundancy analysis (RDA) as determined by 4999 Monte Carlo permutations under the full multivariate model at the three taxonomic levels.

	OTU		Order		Phylum	
Variable	P	F	P	F	P	F
**altitude**	0.002	2.37				
**pH**	0.002	1.68	0.002	1.41		
**moisture**	0.002	1.53	0.002	1.43		
**dust associated bacteria**	0.002	1.32				
**surface patchiness**	0.002	1.28	0.044	1.24	0.002	1.78
**slope**	0.002	1.2				
**C: N**			0.002	2.69	0.002	4.6
**% coarse sand**			0.002	1.45		
**%N**			0.038	1.16		
**habitat type**			0.002	1.14	0.002	1.48
**plant presence**			0.002	1.06		
**% fine sand**					0.002	1.6

Only the relevant environmental factors are listed.

Rugged surface and sorted patterned characteristics shape snow deposition and translocation patterns on slopes affecting the freeze-thaw mediated melt water run-off. The interplay of these parameters with altitude and slope was shown to results in highly diverse and complex local gradients of temperature (>10°C m^−1^; [36]; [43]), moisture content [37]; [43], solar irradiance [35]; [44] and dust deposition rates [11].

### Effects of Time Span and Season

The minimal core microbial community at 6000 m through time, season and space (n = 11 samples) was represented by Alphaproteobacterial genus members *Bradyrhizobium, Nitrobacter* and *Afipia*, whose representatives are involved in nitrogen fixation, nitrification and the use di-methyl sulfone as carbon source, respectively. Their presence implies the existence or development of mechanisms to overcome substrate limitation, UV, freeze-thaw, desiccation and oxidation stress to survive in the high-altitude soils taking advantage of aerosol chemistry, as in the case of *Afipia*, an organism commonly found in the clouds and the atmosphere [45]. The same organisms formed the minimum core community when separately considering the seasonal or temporal component.

**Figure 5:**
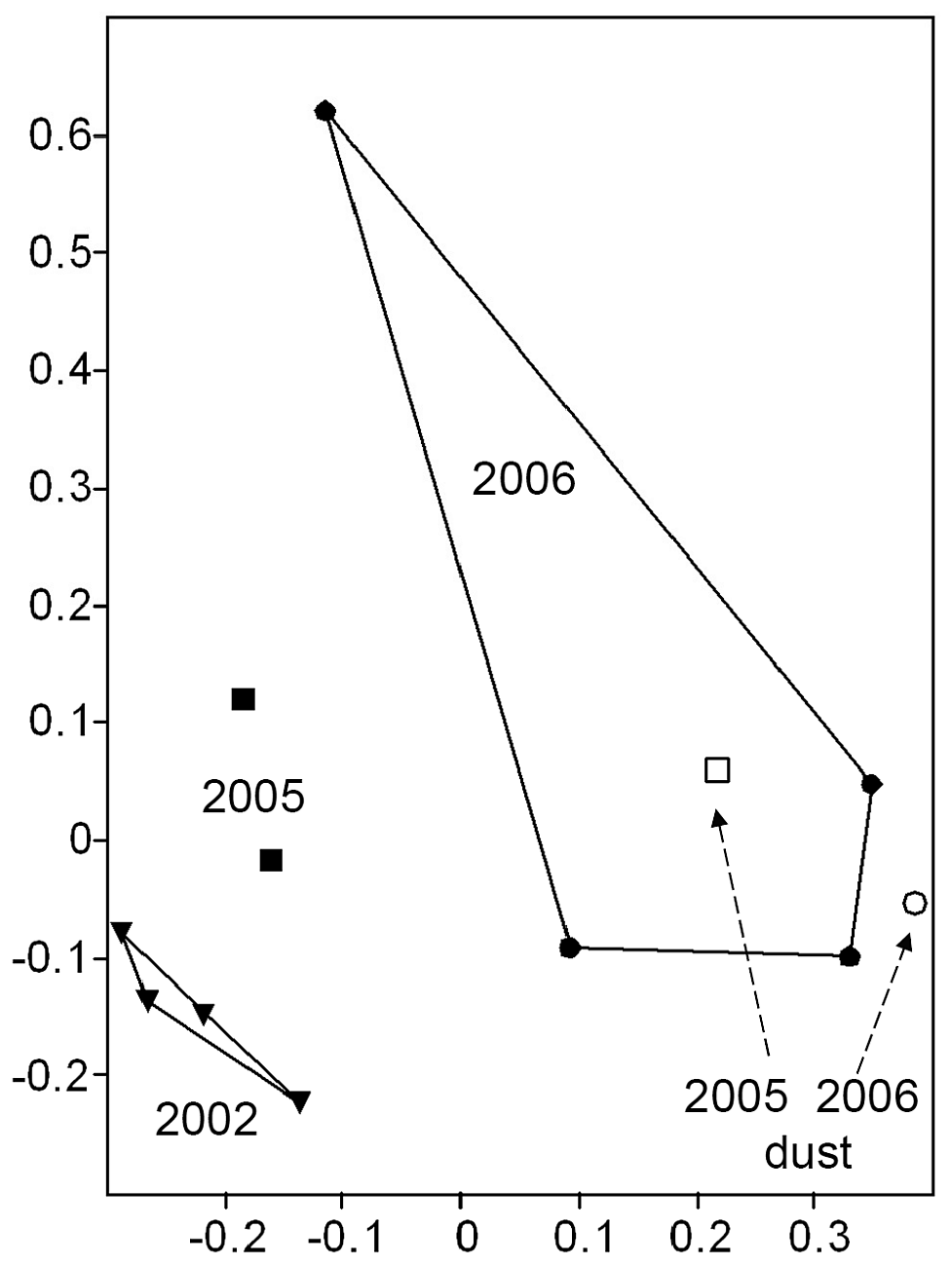
Non-metric multidimensional scaling ordination of 6000 m microbial communities sampled in autumn of 2002 (▾), 2005 (▪) and in spring 2006 (•) next to corresponding 2005 autumn (□) and spring 2006 (○) dust samples. Note - no significant groups could be identified using NP-MANOVA (0.05<p<0.12). Samples from the same group were connected by lines and represent convex hulls of NM-MDS scores of microbial communities as a measure of community-space covered.

Parsimony and Unifrac tests identified significant seasonal and temporal differences (p<0.05) in pairwise comparisons of community structure of (i) seasonal 6000 m transect samples (autumn 2005 vs. spring 2006) and (ii) 3 year deglaciation period in 6000 m transect samples (autumn 2002 vs. autumn 2005). High within group variance in the two deglaciation period groups of samples and also in the two seasonal groups resulted in nearly significant differences in microbial community structure (NP-MANOVA, p = 0.084; p = 0.11, respectively) (Figure 5).

**Figure 6:**
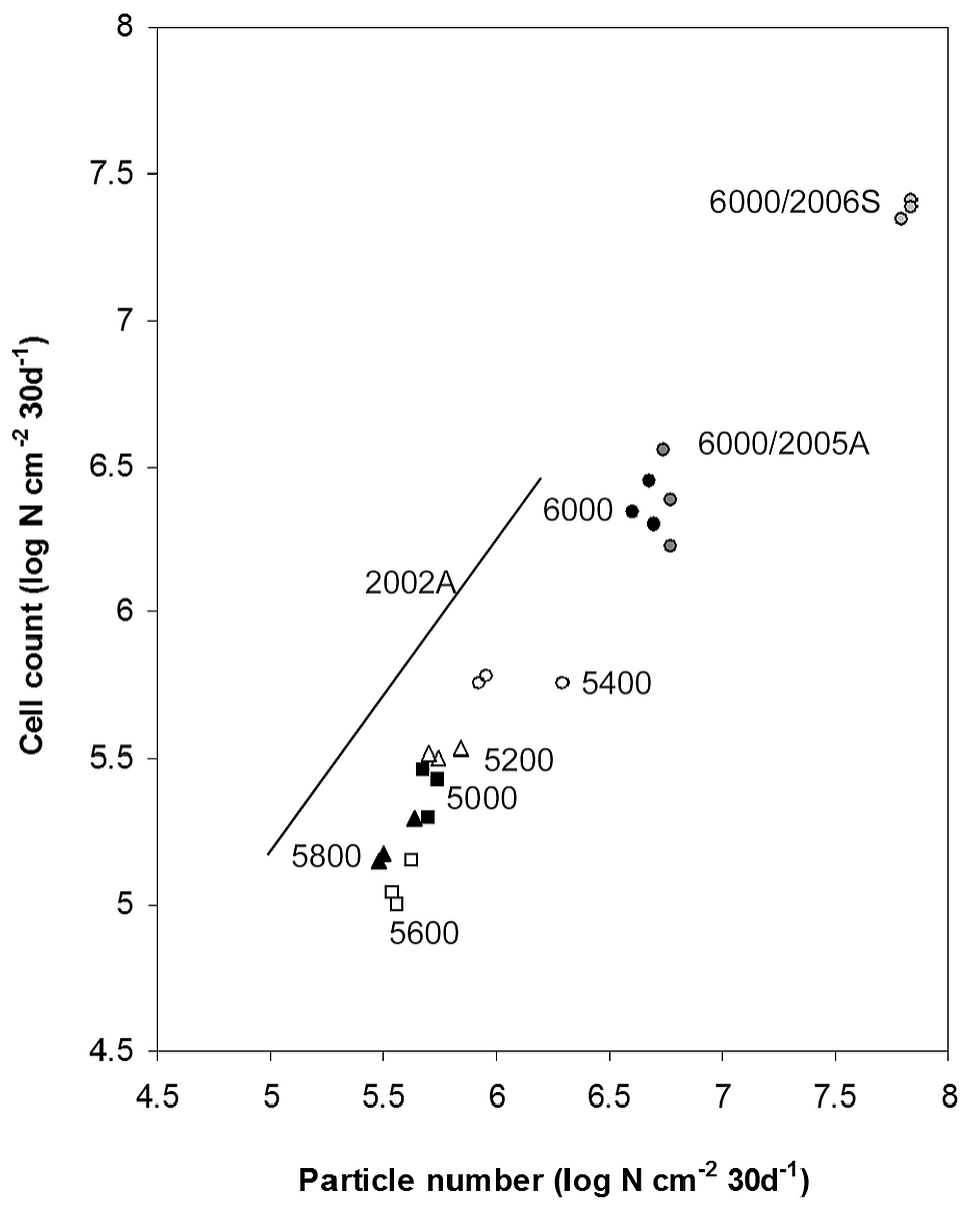
The relationship between dust particle number and microbial abundance during 2002, 2005 and 2006 sampling campaigns. (•) 6000 m, (▴) 5800 m, (□) 5600 m, (○) 5400 m, (?) 5200 m, (▪) 5000 m. (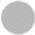) 6000 m autumn 2005; (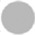) 6000 m spring 2006.

The lack of significant differences is less surprising as almost 40% of all unique OTUs in this study were derived from 6000 m soil and also dust samples from 2005 and 2006. In addition, dust associated bacteria deposition rates were significantly associated (p<0.002) with bacterial community structure in variance partitioning (Table 1). The 6000 m site experienced an order of magnitude higher dust deposition rates than other sites (Figure 6). Particle and bacterial cell deposition rates were an additional order of magnitude higher in 2006 spring samples due to premonsoon ramp up-effect [8]; [35] and coincided with the orders of magnitude higher within group sum of squares in NP-MANOVA of spring 6000 m soil communities (Figure 5; Figure 6).

The nearly significant differences in 6000 m microbial communities in response to a real 3-year deglaciation period contradicted the results of a 4-year deglaciation chronosequence at 5000 m in the Andes [39]. Also, the relatively low representation of taxa linked to primary production like *Cyanobacteria* (<0.5%) in comparison to 12% or 31% or higher in the Andean 5000 m deglaciated soil [39] delineated the two habitats. In our study, *Cyanobacteria* distribution could not be linked to any measured environmental parameter or season, however their low abundance was consistent with an almost three orders of magnitude decrease in phototroph abundance in Yala glacier in the Himalaya from 5000 to 5500 m [43] and reflected a 20% increase in UV AB irradiation per 1000 m starting at a UV-B dose of 4.5 W m^−2^ at 4517 m [44]. Consequently, previously observed microbial activities in the Himalayan glacier surface (5000 m) or barren (5800 or 6000 m) soils [18] are more likely driven by dust associated organic fraction (>40% of dust mass fraction; [8]) than primary production [46]. The orders of magnitude higher dust deposition rates [47]; [48] distinguish the Himalayan sites from the Andean and point to the imminent role of tropospheric dust on microbial community structure and succession.

### Dust, Microbial Communities and Viability

Particle size diameter measurements showed a monomodal distribution (d = 4–12 µm) that was not significantly different (p>0.05) between seasons. The lack of large particles (d>20 µm) indicated that the dust was not directly from the site itself [11]. Microscopy revealed that the fraction of bacterial cells represented from 23±5% to 36±12% of the total deposited dust particles and within the characteristic limits observed recently for high troposphere [43]; [49]; [50] and also high-altitude glacier ice samples [51]. A tight relationship (R^2^ = 0.92 and R^2^ = 0.97) between the deposited particle and particle-associated bacterial abundance was observed in 2002 and aggregated dust trap datasets, respectively (Figure 6). The relationship between dust (and bacterial) deposition in relation to altitude was not linear (Figure 6) in line with site patchiness (Figure S5 in File S1) and the general complexity of dust deposition patterns [11]. Autumn dust deposition rates were an order of magnitude higher at the 6000 m site than other sites (Figure 6). Quantitative differences between autumn and spring dust deposition rates at 6000 m coincided with low and high deposition periods described before [8]; [35]. Autumn deposition rates observed in this study were thus rather conservative or at best intermediate estimates of monthly dust deposition rates (Figure 6) [8]; [35].

The microbial groups detected in 6000 m dust samples deposited at the middle troposphere altitude included essentially all microbial groups recently described for high-troposphere aerial samples [45] with the exception of *Planctomycetes*, suggesting their tropospheric origin. The relative and dynamic differences in the composition of tropospheric microbiome described recently [45] were reflected also in the dust microbiota samples in this study illustrating the very dynamic and diverse nature of this system. Pairwise parsimony and Unifrac tests identified significant differences (p<0.05) in the structure of microbial communities from (i) seasonal 6000 m dust trap samples of autumn 2005 vs. spring 2006; (ii) dust trap vs. 6000 m 2002, 2005 and 2006 soil samples; (iii) dust trap vs. samples collected at other altitudes. The autumn 2005 dust sample OTUs were also detected in spring 2006 soil samples and the removal of all OTUs detected in 6000 m dust samples from 2005 and 2006 soil datasets increased the soil community similarity (soil samples from 2005 vs. 2006) from 0.85±0.07 to 0.94±0.03. The fact that dust associated OTUs’ presence decreased the apparent recipient soil community similarity was also evident from lower similarities between soil samples and soil samples devoid of corresponding year dust OTUs (autumn 2005 (0.93±0.03); spring 2006 (0.92±0.04)). These results show that OTUs from tropospheric dust associated microbial communities have the potential to influence the observed relationships between the recipient soil communities. In addition, dust associated bacteria were recently shown to be viable [45]; [49]; [50] as was also shown in our low temperature cultivation experiment reported below.

Finally, inspection of our custom database entries in full format GenBank file revealed that the physical origin of the most closely related sequences to dust trap OTU sequences spanned practically all habitats: marine, terrestrial, lacustrine, arctic, alpine, temperate, water, air, soil and sediment, suggesting a broadly distributed origin of incoming dust bacteria. The HYSPLIT model back trajectories computed for the duration of all sampling events (2002, 2005, 2006 and 2007 (Table S1 in File S1)) and MODIS dust data supported this observation (Figure S7 in File S1 and Figure S8 in File S1, respectively), identified the source regions as spanning from Sahara to Bay of Bengal and from Caspian Sea to Tibet and indicated significant differences in percentage distributions of source regions between sampling campaigns (Table S3 in File S1).

Dust particle size distributions in prior long-range dust studies [52]; [53]; [54] were not significantly different from those observed in our dust traps (d = 4–12 µm) and snow samples (d = 3–10 µm). This shows that a portion of the dust associated cells observed in our study traveled the long distances (d>1000 km) before deposition [35]; [55]; [56]. Underestimation of the size of the global dust cycle was established implying that the deposition flux of dust and its fertilizing effects on ecosystems are substantially larger than thought before [19]. Massive long range dust deposition data reevaluated recently resulted in almost tenfold increase in the reported dust fluxes to the Himalaya [48], establishing the missing coupling between high-altitude soil and tropospheric deposits.

As aerial bacteria were shown to be abundant [45]; [49]; [50]; [57] extensively covered with mucus like material [58], metabolically active [43]; [50] and viable at low temperatures [43]; [59] it appears that short HYSPLIT transit times (t<4 days) and dust deposition patterns observed in this study determine the initial biomass pool and incoming species composition. In addition, recent study on global dust deposition identified biological aerosols as ice-nuclei playing an important role in snow precipitation [60] and shaping environmental characteristics at deposition sites. Bacterial abundance in our autumn (3 ·10^4^±0.6 cells mL^−1^) and spring (5 ·10^4^±0.9 cells mL^−1^) fresh surface snow samples (6000 m) was within the ranges reported before for the Himalayan surface snow [61] and remote high-altitude glacier lakes [62].

We cultivated psychrotolerant microorganisms from these deposits that exhibited growth in tight clusters resembling cell clumps associated with particles in unfixed snow samples (n = 9; not shown). The viability of dust-associated bacteria entering our 6000 m site was in-line with recent reports on high viability of bacterial cells (16–48%) in tropospheric samples from the Gulf of Mexico [45] and from Asian dust reaching Japan [49]; [50] and North America [57]. The growth potential of deposited microbial cells is further modified by the severe high-altitude postdeposition selection processes [63]. Our recent observation showed that the high-altitude environment effectively selected for a minor (<10%) but resistant portion of bacteria from unadapted soil communities [18], providing the surviving microbiota with C and N from the annihilated biomass [18] in addition to resources from ancient dust, minerals [46], [51] and substantial ongoing deposits [19]; [48]; [64] from troposphere. Our results indicate that deposited particle associated cells are important for the bacterial community assembly and geochemistry at the high-altitude surface-atmosphere interphase.

## Conclusions

The major findings of this study can be summarized as:

1. Complex gradients in soil and environmental characteristics give rise to highly variable environmental niches on topographically complex high-altitude slopes. Consequently, phylogenetically close or distantly related communities were equally likely at short distances and no significant differences in microbial community structure were observed between groups of samples in response to season or time since deglaciation.
2. Dust microbial community samples differed significantly and differed also from recipient soils. Closest relatives of dust OTUs, HYSPLIT back-trajectory calculations and dust particle sizes all supported that the deposited microbes came from long distances. Detection of viable microbes in the dust is consistent with the short transit times of tropospheric dust.
3. An order of magnitude lower fraction of *Cyanobacteria* in the Himalayan soils points to the importance of tropospheric dust and aerosol chemistry as sources of nutrients to microbial communities in the 6000 m recently deglaciated environments.
4. Although deposition rates were highly variable with altitude, a tight relationship existed between dust and associated cells. The coincidence of quantitative differences between autumn and spring dust deposition rates at 6000 m with low and high deposition periods described before showed that the autumn dust deposition rates were more conservative or at best intermediate estimates of monthly dust deposition rates.
5. The spatial, environmental and temporal complexity of the high-altitude soils of the Himalaya generates ongoing disturbance and colonization events that subject heterogeneous microniches to stochastic colonization by dust associated microbes and results in the observed spatially divergent bacterial communities.

## Supporting Information

File S1 **Electronic supplementary material.** **Figure S1A:** The location of Drohmo Peak sampling site in relation to recent reports on aerosol chemistry and dynamics in its vicinity (Carrico et al., 2009; Chatterjee et al., 2011). The inset shows a wider geographical orientation of the map. The Nepal Climate Observatory-Pyramid (NCO-P) at the foothills of Mt. Everest at 5079 m, is a comparable location to our site and provided a systematic 2-year measurement of dust concentrations and composition (Decesari *et al.*, 2009). An experimental station in Darjeeling (2194 m) provided evidence that 33% and 20% of particles deposited at much lower altitude and in populated and agriculturally intensive area originated from long-distance (>1500 km) continental and marine sources, respectively (Chatterjee et al., 2011). Landsat image data were obtained from USGS EROS (Earth Resources Observatory and Science (EROS) Center) public domain (http://eros.usgs.gov/#) using LandsatLook Viewer software (http://landsatlook.usgs.gov/), courtesy of U.S. Geological Survey, Department of the Interior/USGS, U.S. Geological Survey. The USGS home page is http://www.usgs.gov. **Figure S1B:** A satellite image of the rugged surface topography surrounding Drohmo peak (6980 m), Nepal (27° 48′ 00″ N and 88° 07′ 02″ E) and the sampling site (circled). Image courtesy of the Image Science & Analysis Laboratory, NASA Johnson Space Center (http://eol.jsc.nasa.gov) unique photo number (Mission-Roll-Frame): ISS008-E-06645. **Figure S1C:** The view of the sampling site from the lower Kanchenjunga glacier valley to the East, from a distance of 8 km. **Figure S1D:** High-altitude sampling sites. Photo: Blaž Stres. **Figure S2:** Sampling scheme. Six altitude transects on the south facing slope were established. Each transect was 150 m in length, every 200 m difference in altitude. Four sampling sites were spaced horizontally at 50 m. Soil cores (red asterix) were taken at each sampling site (red circle). **Figure S3:** The 3D representations of the measured environmental parameters in this study. Ts- each of the four transect sites per altitude transect. (A) soil carbon content, (B) soil nitrogen content, (C) reducing sugars glucose equivalents, (D) coarse sand, (E) fine sand, (F) coarse silt, (G) fine silt, (H) clay, (I) extractable ammonia content, (J) extractable nitrate content, (K) MWI - molecular weight index of extractable water soluble organic carbon, (L) pH. **Figure S4:** An overview of gradients in measured environmental parameters that were significantly associated (p<0.05) to bacterial community structure in the high-altitude soils at 97% OTU level. For spatial interpolation spherical, exponential, Gaussian and cubic models were tested, semivariograms were constructed using cubic model, cross validated by jackknifing after which kriging algorithm was used. (A) pH; (B) moisture; (C) dust bacteria deposition rates; (D) patchiness; (E) slope; (F) sugar content (p = 0.08). Spatial complexity of gradients was projected to the vertical axis spanning 1000 m altitude gradient from 5000 m to 6000 m. **Figure S5:** An example of the landscape scale surface curvature as determined using Arc-GIS using merged profile curvature and plan curvature. The slope governs the overall rate of movement downwards. Aspect defines the direction of flow. The profile (i.e. horizontal) curvature affects the acceleration and deceleration of flow and, therefore, influences erosion and deposition. The plan (i.e. vertical) curvature influences convergence and divergence of flow. Considering both plan and profile curvature together allows us to understand more accurately the flow of water (snow-meltwater runoff), snow (avalanche) and unidirectional air masses across a surface (http://authors.library.caltech.edu/25021/2/cabook.pdf). **Figure S6:** NM-MDS ordination of bacterial microbial communities sequenced from 142 samples of **temperate**, **volcanic**, **Himalayan**, **Antarctic**, **Spitzbergen** permafrost**, marine**, **freshwater (lake and river)** and **contaminated** soils and sediments [21]. **Figure S7:** An example of characteristic (A) autumn and (B) spring trajectories of air masses during the dust sampling and snow collection campains. HYSPLIT_4 (Hybrid Single Particle Lagrangian Integrated Trajectory; http://ready.arl.noaa.gov/HYSPLIT.php) Model access via OAA Air Resources Laboratory, Silver Spring, USA (http://ready.arl.noaa.gov) was used to compute back trajectories for all sampling experiments at 00.00 site local time. **Figure S8:** Average monthly aerosol amounts around the world based on observations from the Moderate Resolution Imaging Spectroradiometer (MODIS: http://modis.gsfc.nasa.gov/) on NASA’s Terra satellite (http://terra.nasa.gov/). An optical thickness of less than 0.1 (palest yellow) indicates a crystal clear sky with maximum visibility, whereas a value of 1 (reddish brown) indicates very hazy conditions. Source: NASA’s Earth Observatory (http://earthobservatory.nasa.gov/). **Table S1:** Schematic representation of the sample distribution, sampling seasons and analyses performed in this study: (**▪**/_/_/_) physical-chemical analyses of soil characteristics; (_/**▪**/_/_) pyrosequencing; (_/_/_**▪**/_) direct particle and bacterial cell enumeration, size estimation; (_/_/_/**▪**) cultivation experiment at 4°C. Numbers represent number of sampling sites analyzed. See materials and methods for details. **Table S2:** Sequencing coverage of samples from the three sampling periods (Table S1). **Table S3:** The percentage distribution of source regions were determined based on the ratio of the number of events of the regions to the total number of sampling events as calculated using HYSPLIT_4 model developed by NOAA/ARL. Back trajectories to source regions were computed for all sampling events on daily basis and expressed as relative distribution.
(PDF)
