# Correction: Recently Deglaciated High-Altitude Soils of the Himalaya: Diverse Environments, Heterogenous Bacterial Communities and Long-Range Dust Inputs from the Upper Troposphere

**DOI:** 10.1371/annotation/1406824c-f082-400b-8fc7-4995c97a6249

**Published:** 2014-01-14

**Authors:** Blaz Stres, Woo Jun Sul, Bostjan Murovec, James M. Tiedje

An error was introduced in the preparation of this article for publication. In Figure Legend 6, ∆ was replaced with a question mark. The correct version of Figure Legend 6 can be viewed here: http://www.plosone.org/corrections/pone.0076440.001.cn.tif

