# Supplementary material for: Recently Deglaciated High-Altitude Soils of the Himalaya: Diverse Environments, Heterogenous Bacterial Communities and Long-Range Dust Inputs from the Upper Troposphere
Source: PLoS One. 2013 Sep 26;8(9):e76440. doi: 10.1371/journal.pone.0076440 (PMC3784432; doi:10.1371/journal.pone.0076440)
Supplement: File S1 — Electronic supplementary material. Figure S1A: The location of Drohmo Peak sampling site in relation to recent reports on aerosol chemistry and dynamics in its vicinity (Carrico et al., 2009; Chatterjee et al., 2011). The inset shows a wider geographical orientation of the map. The Nepal Climate Observatory-Pyramid (NCO-P) at the foothills of Mt. Everest at 5079 m, is a comparable location to our site and provided a systematic 2-year measurement of dust concentrations and composition (Decesari et al., 2009). An experimental station in Darjeeling (2194 m) provided evidence that 33% and 20% of particles deposited at much lower altitude and in populated and agriculturally intensive area originated from long-distance (>1500 km) continental and marine sources, respectively (Chatterjee et al., 2011). Landsat image data were obtained from USGS EROS (Earth Resources Observatory and Science (EROS) Center) public domain (http://eros.usgs.gov/#) using LandsatLook Viewer software (http://landsatlook.usgs.gov/), courtesy of U.S. Geological Survey, Department of the Interior/USGS, U.S. Geological Survey. The USGS home page is http://www.usgs.gov. Figure S1B: A satellite image of the rugged surface topography surrounding Drohmo peak (6980 m), Nepal (27° 48′ 00″ N and 88° 07′ 02″ E) and the sampling site (circled). Image courtesy of the Image Science & Analysis Laboratory, NASA Johnson Space Center (http://eol.jsc.nasa.gov) unique photo number (Mission-Roll-Frame): ISS008-E-06645. Figure S1C: The view of the sampling site from the lower Kanchenjunga glacier valley to the East, from a distance of 8 km. Figure S1D: High-altitude sampling sites. Photo: Blaž Stres. Figure S2: Sampling scheme. Six altitude transects on the south facing slope were established. Each transect was 150 m in length, every 200 m difference in altitude. Four sampling sites were spaced horizontally at 50 m. Soil cores (red asterix) were taken at each sampling site (red circle). Figure S3: The 3D representations [file pone.0076440.s001.pdf]

ELECTRONIC SUPPLEMENTARY MATERIAL

Title:

Recently deglaciated high-altitude soils of the Himalaya: diverse environments, heterogenous bacterial communities and long-range dust inputs from the upper troposphere

Contributors: Blaž Stres[1], Woo Jun Sul[2,3], Boštjan Murovec[4], James M. Tiedje[2]*

Complete postal addresses:

[1]Department of Animal Science, University of Ljubljana, Domzale, Slovenia;

[2] Center for Microbial Ecology, Michigan State University, East Lansing, Michigan, USA;

[3]Department of Systems Biotechnology, Chung-Ang University, Anseong, Korea;

[4] Faculty of Electrical Engineering, University of Ljubljana, Ljubljana, Slovenia;

[*]*Corresponding author:* James M. Tiedje, Center for Microbial Ecology, Michigan State University, Plant and Soil Science Building 540, East Lansing, MI48828, USA; e-mail: tiedjej@msu.edu

Content:

I.SUPPLEMENTARY FIGURES 1-8
II. SUPPLEMENTARY TABLES 1-3

I. SUPPLEMENTARY FIGURES

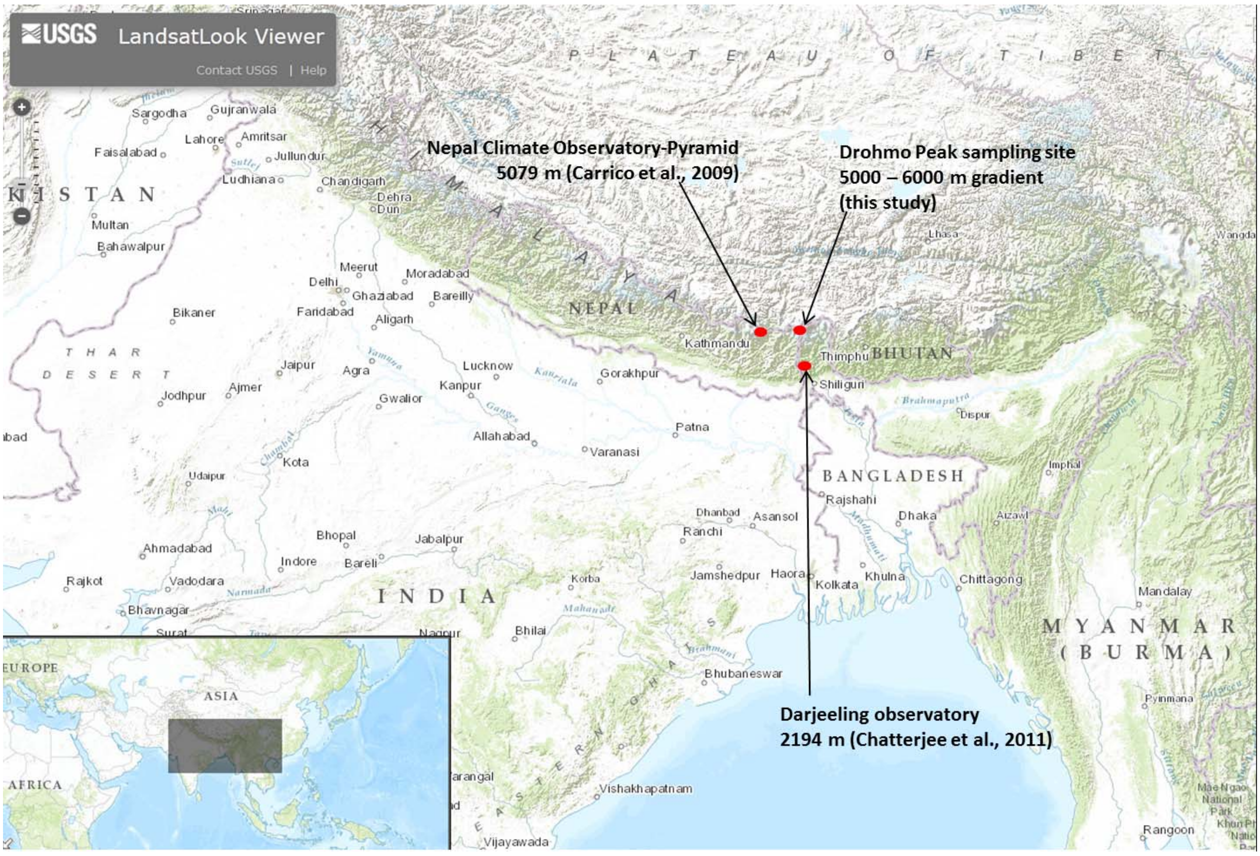

Figure S1A: The location of Drohmo Peak sampling site in relation to recent reports on aerosol chemistry and dynamics in its vicinity (Carrico et al., 2009; Chatterjee et al., 2011). The inset shows a wider geographical orientation of the map.

The Nepal Climate Observatory-Pyramid (NCO-P) at the foothills of Mt. Everest at 5079 m, is a comparable location to our site and provided a systematic 2-year measurement of dust concentrations and composition (Decesari *et al.*, 2009).

An experimental station in Darjeeling (2194 m) provided evidence that 33% and 20% of particles deposited at much lower altitude and in populated and agriculturally intensive area originated from long-distance (>1500 km) continental and marine sources, respectively (Chatterjee et al., 2011).

Landsat image data were obtained from USGS EROS (Earth Resources Observatory and Science (EROS) Center) public domain (http://eros.usgs.gov/#) using LandsatLook Viewer software (http://landsatlook.usgs.gov/), courtesy of U.S. Geological Survey, Department of the Interior/USGS, U.S. Geological Survey. The USGS home page is http://www.usgs.gov.

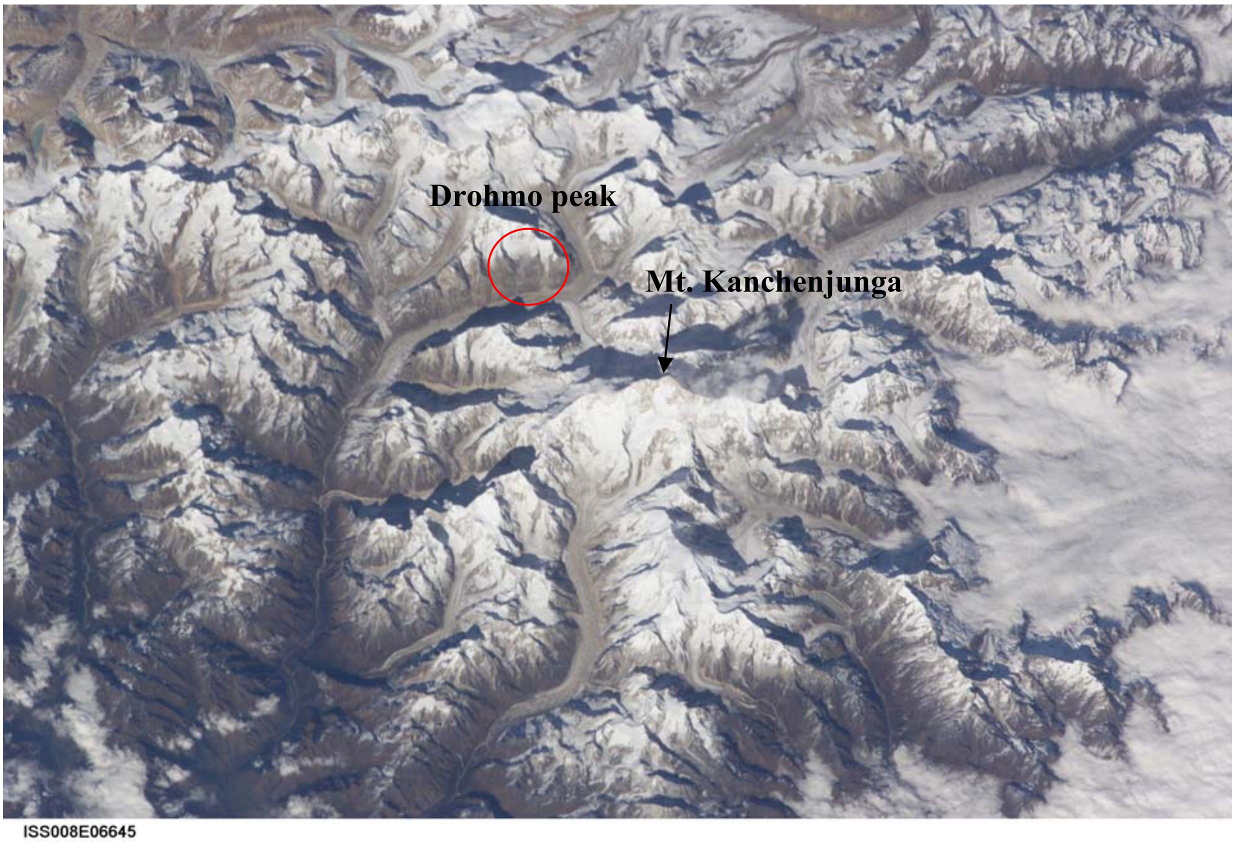

Figure S1B: A satellite image of the rugged surface topography surrounding Drohmo peak (6980 m), Nepal (27° 48' 00'' N and 88° 07' 02'' E) and the sampling site (circled). Image courtesy of the Image Science & Analysis Laboratory, NASA Johnson Space Center (http://eol.jsc.nasa.gov) unique photo number (Mission-Roll-Frame): ISS008-E-06645.

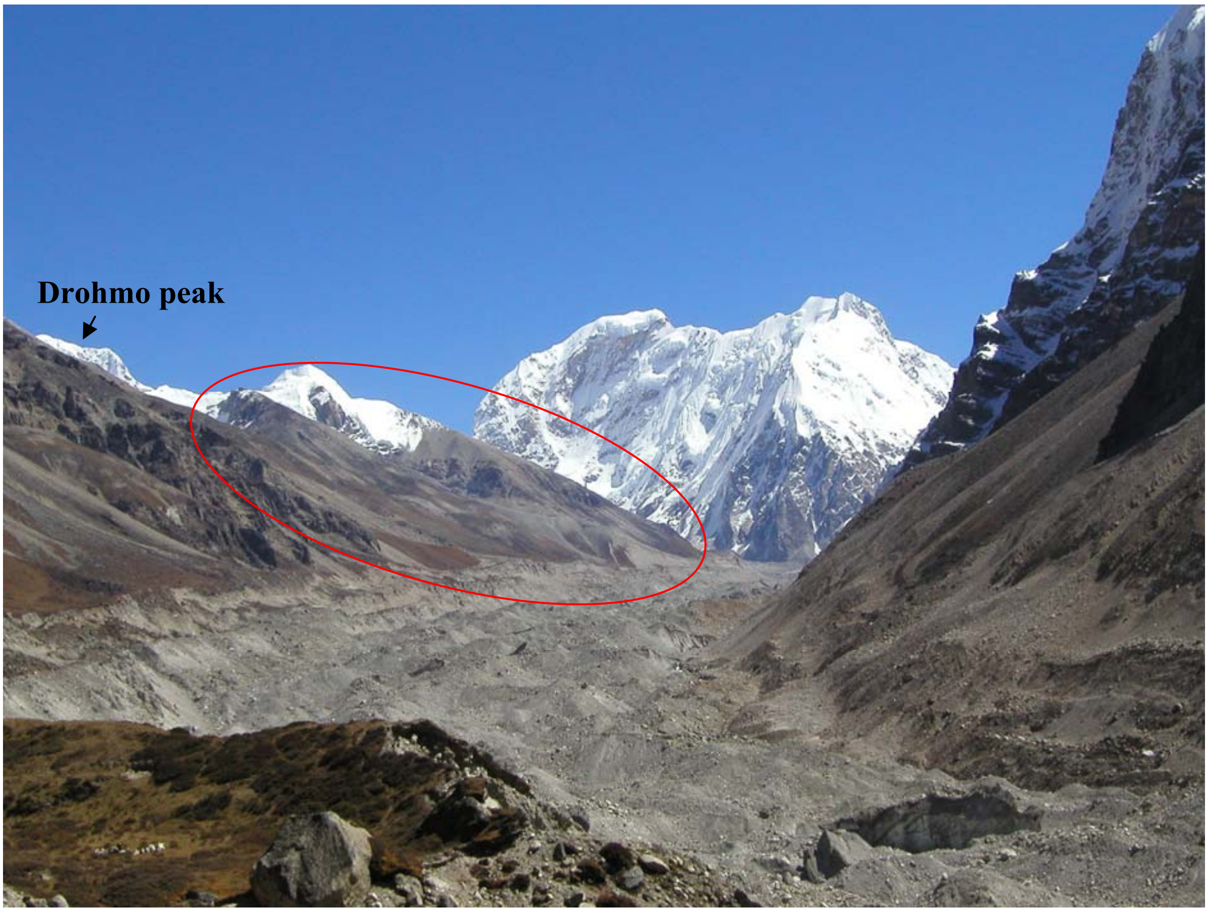

Figure S1C: The view of the sampling site from the lower Kanchenjunga glacier valley to the East, from a distance of 8 km.

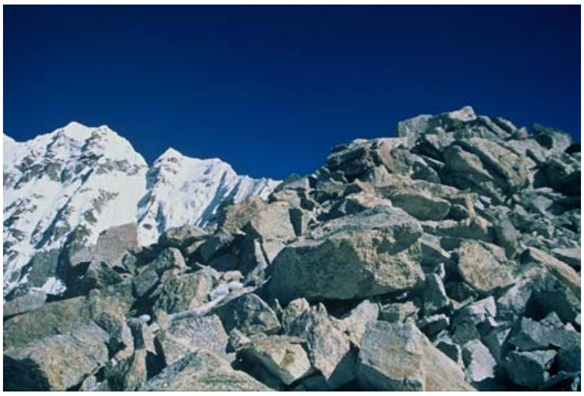

6000 m

Figure S1D: High-altitude sampling sites. Photo: Blaž Stres

5800 m: view upwards (left) and below (right)

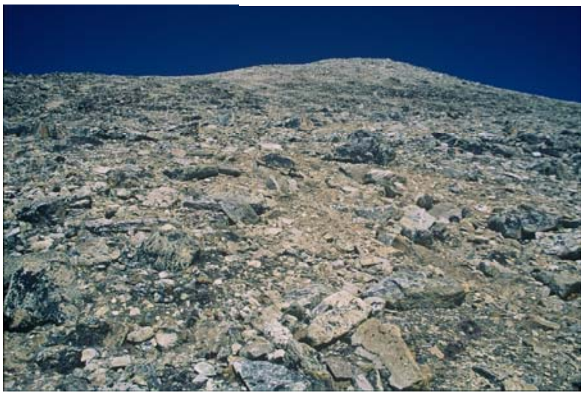

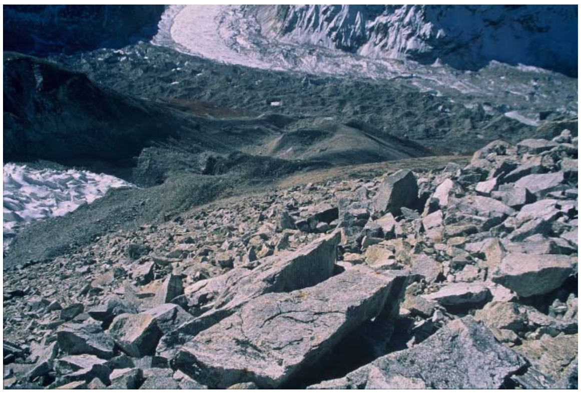

5600m: view upwards (left) and below (right)

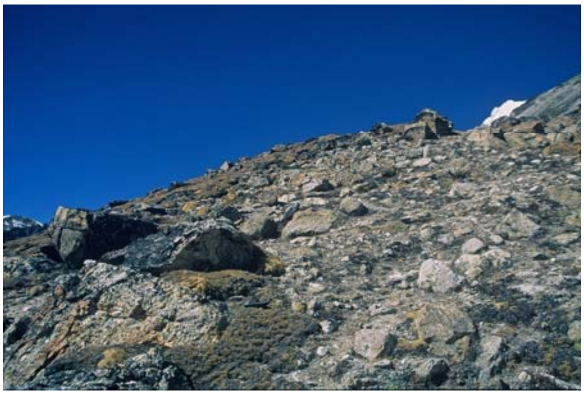

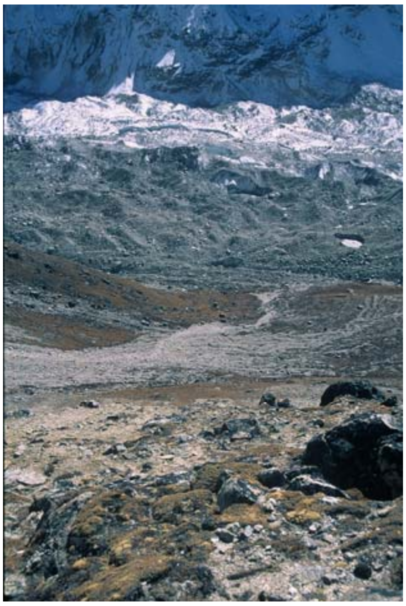

Figure S1D continued

5400 m: view upwards (left) and below (right)

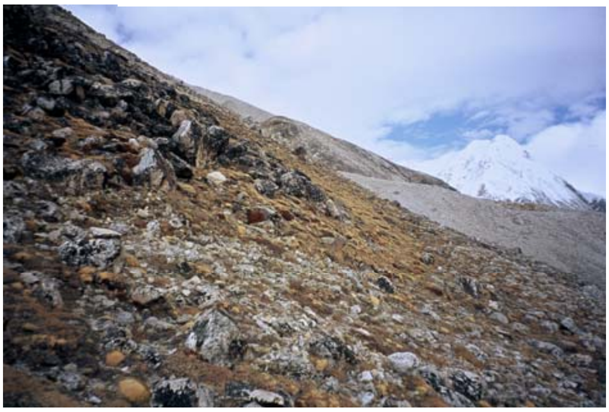

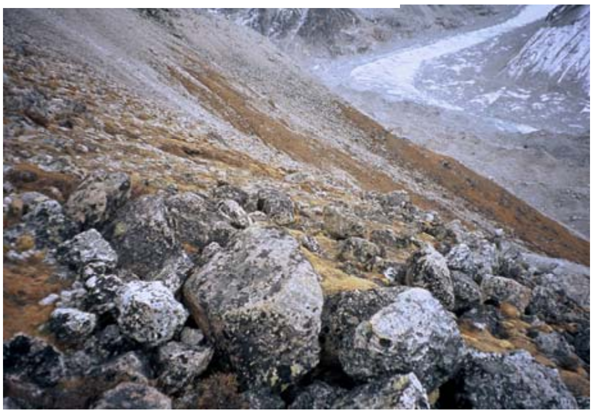

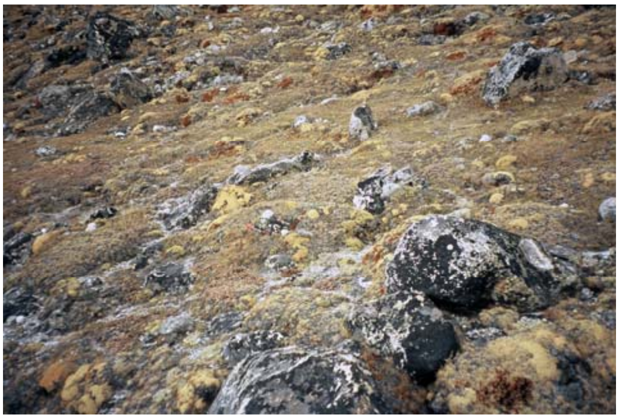

5200 m

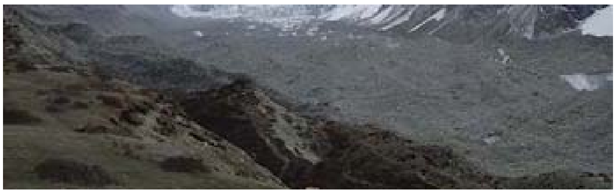

5000 m

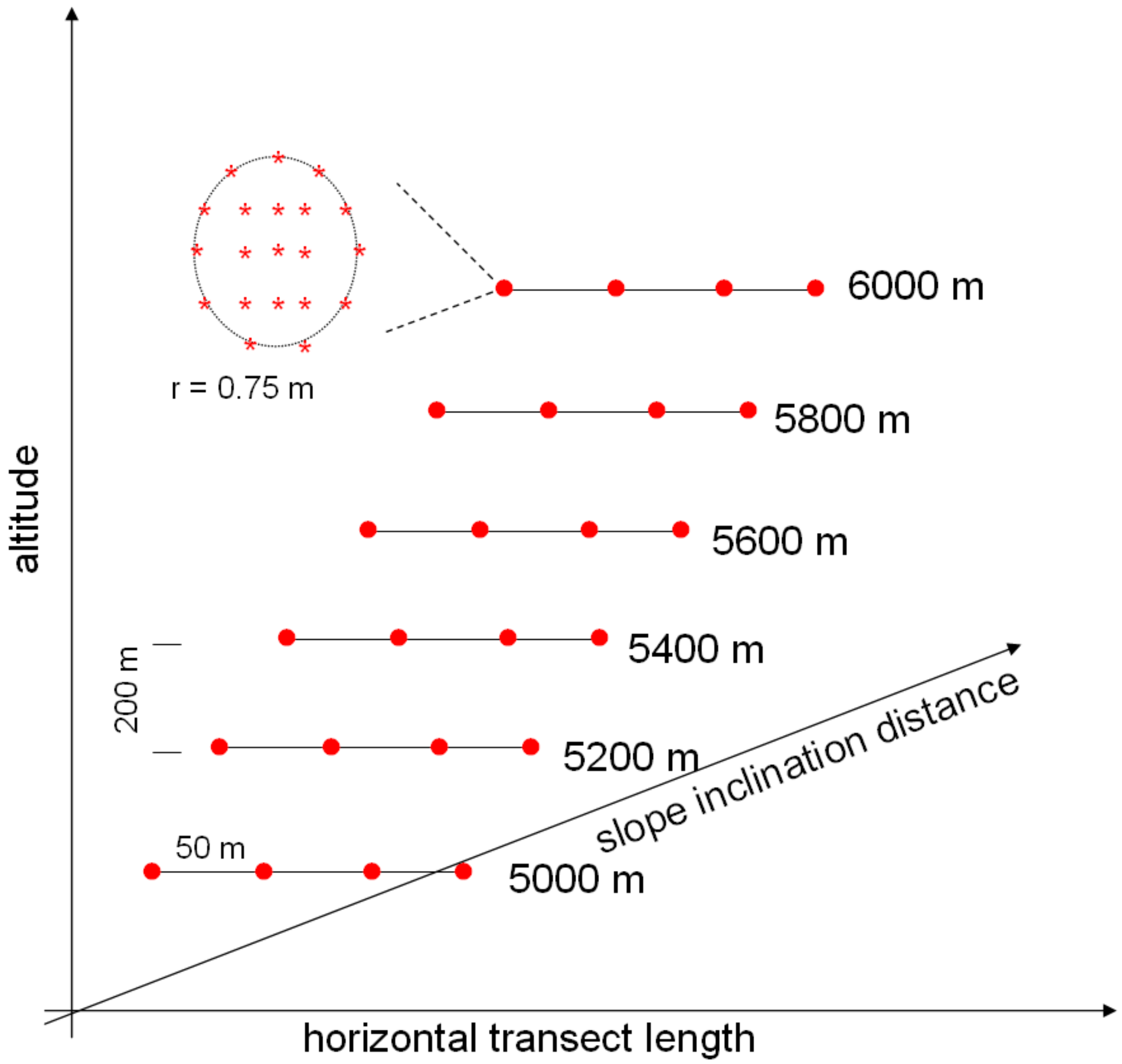

Figure S2: Sampling scheme. Six altitude transects on the south facing slope were established. Each transect was 150 m in length, every 200 m difference in altitude. Four sampling sites were spaced horizontally at 50 m. Soil cores (red asterix) were taken at each sampling site (red circle).

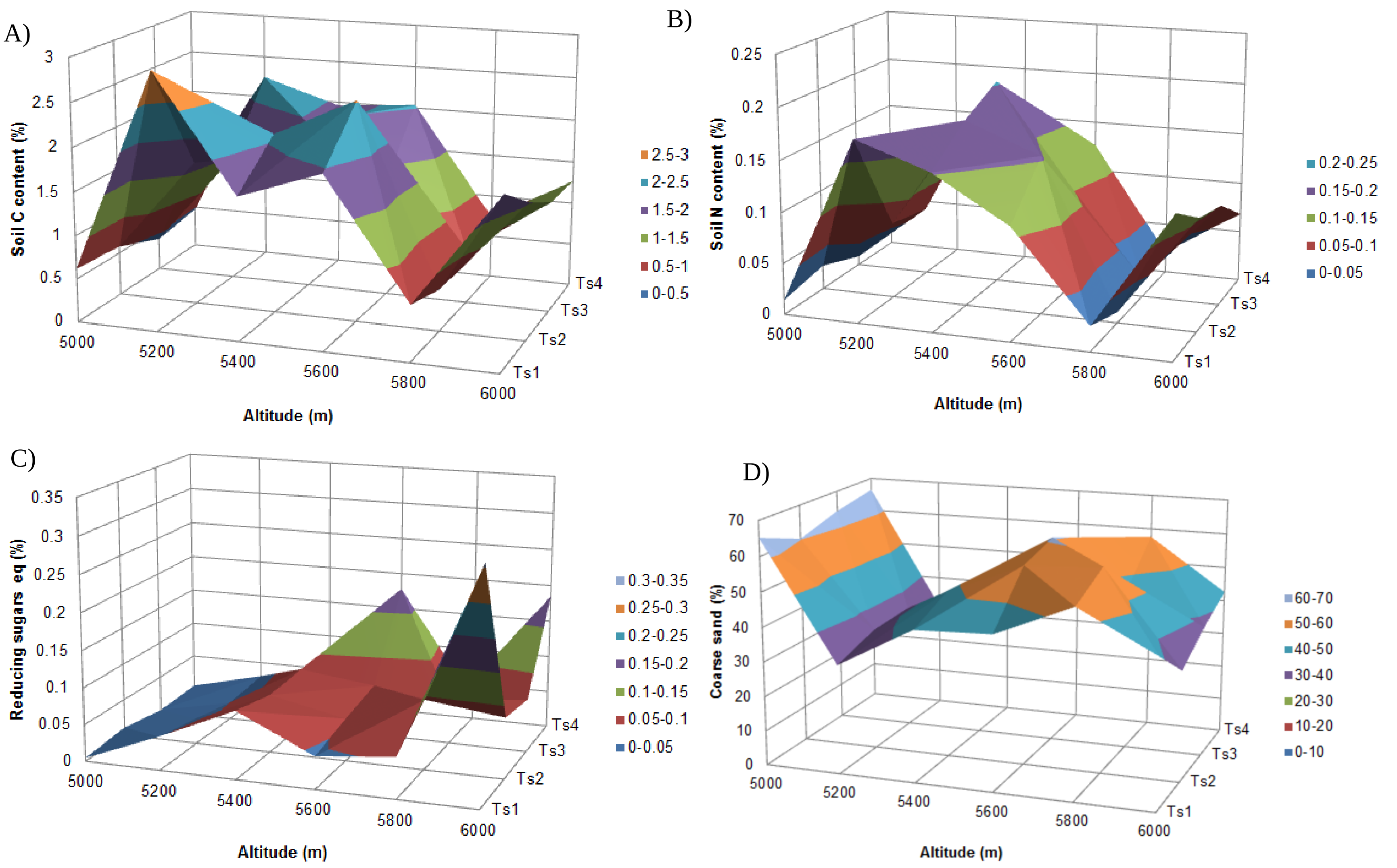

Figure S3 (continued)

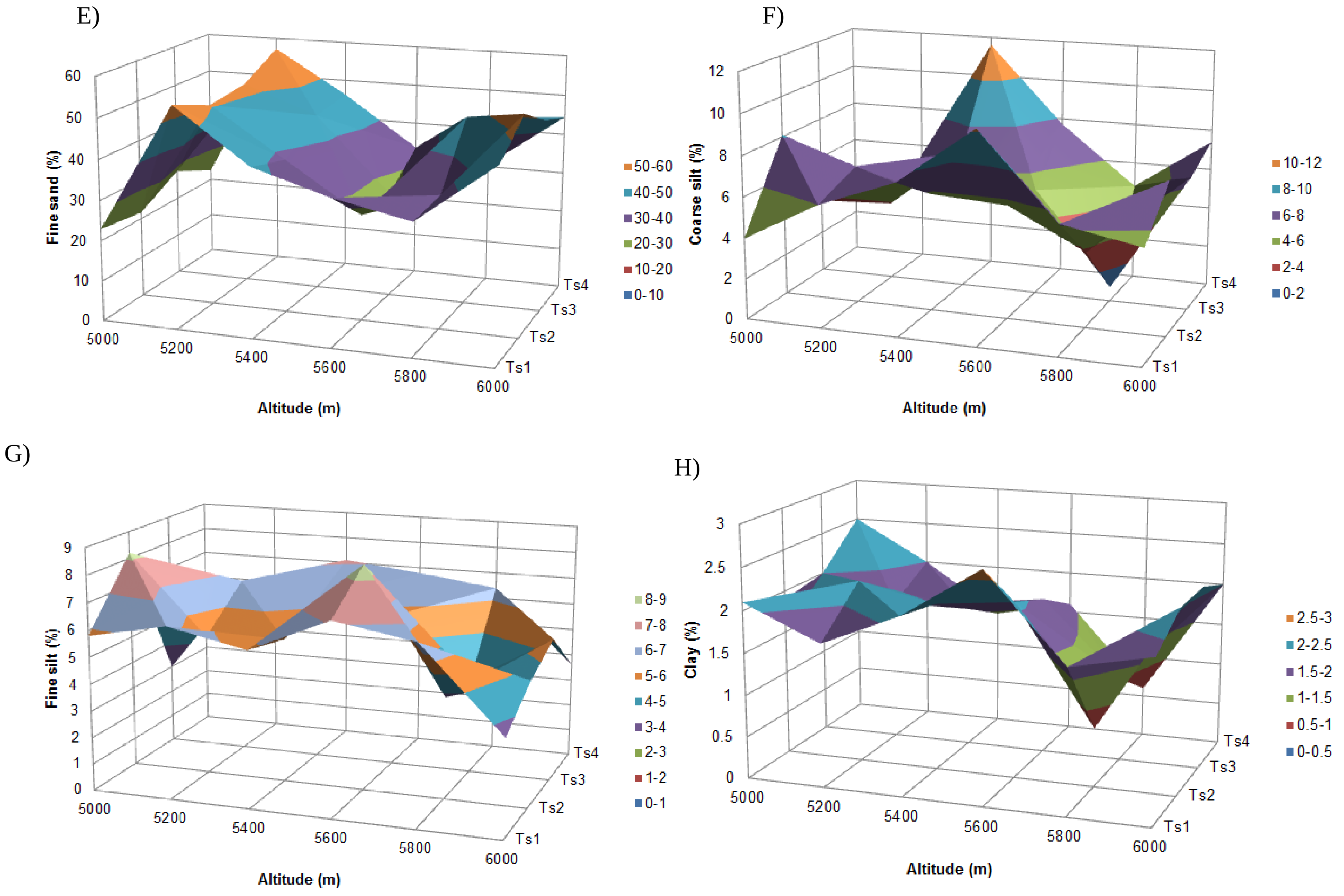

Figure S3 (continued)

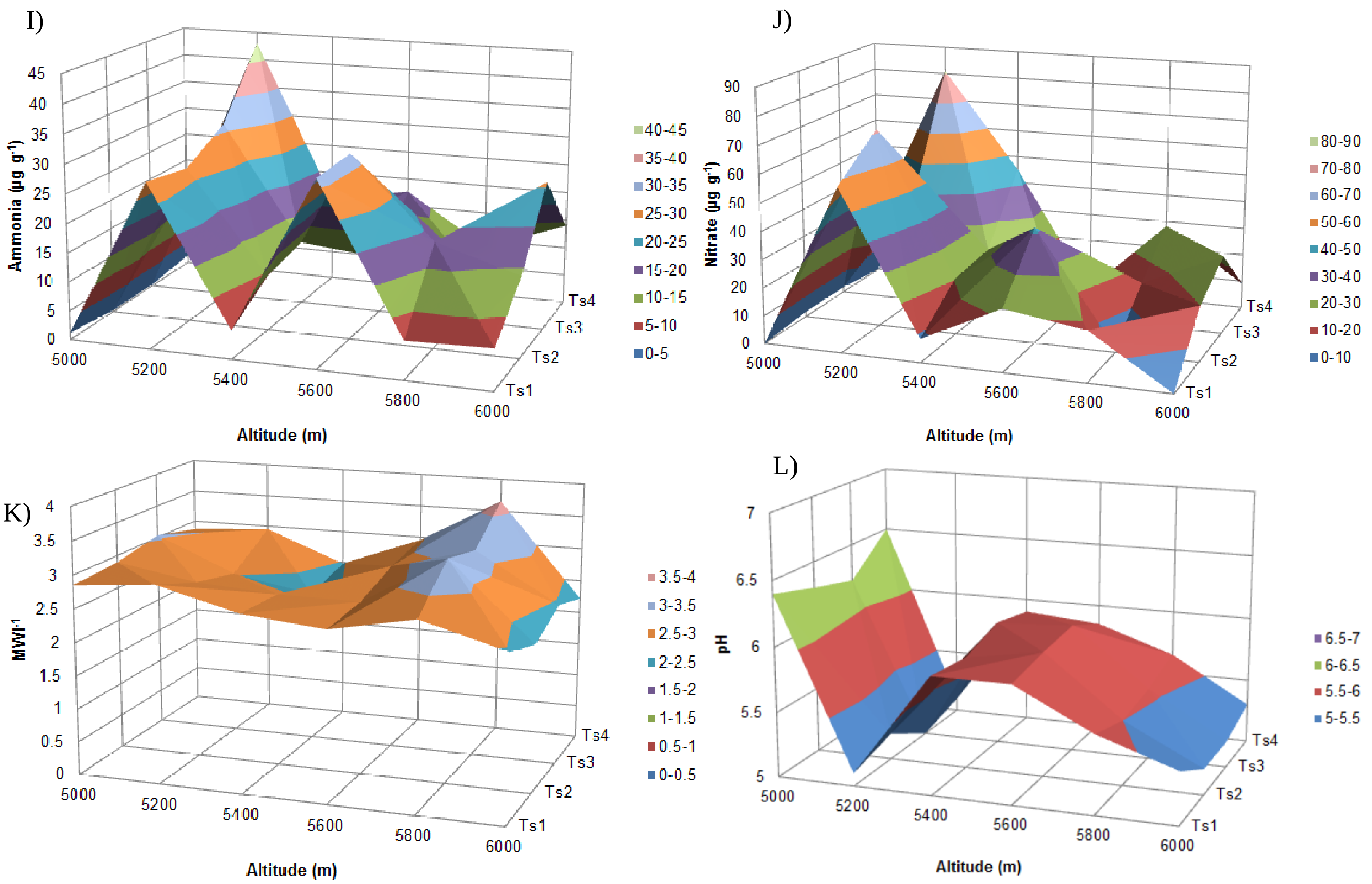

Figure S3: The 3D representations of the measured environmental parameters in this study. Ts- each of the four transect sites per altitude transect. (A) soil carbon content, (B) soil nitrogen content , (C) reducing sugars glucose equivalents, (D) coarse sand, (E) fine sand, (F) coarse silt, (G) fine silt, (H) clay, (I) extractable ammonia content, (J) extractable nitrate content, (K) MWI - molecular weight index of extractable water soluble organic carbon, (L) pH.

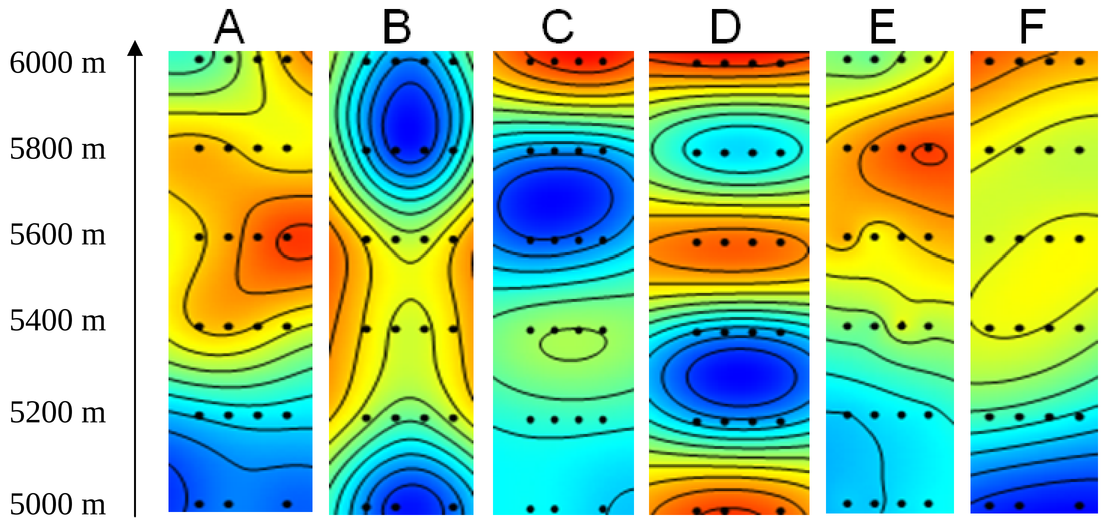

Figure S4: An overview of gradients in measured environmental parameters that were significantly associated ($p<0.05$) to bacterial community structure in the high-altitude soils at 97% OTU level. For spatial interpolation spherical, exponential, Gaussian and cubic models were tested, semivariograms were constructed using cubic model, cross validated by jackknifing after which kriging algorithm was used. (A) pH; (B) moisture; (C) dust bacteria deposition rates; (D) patchiness; (E) slope; (F) sugar content ($p=0.08$). Spatial complexity of gradients was projected to the vertical axis spanning 1000 m altitude gradient from 5000 m to 6000 m.

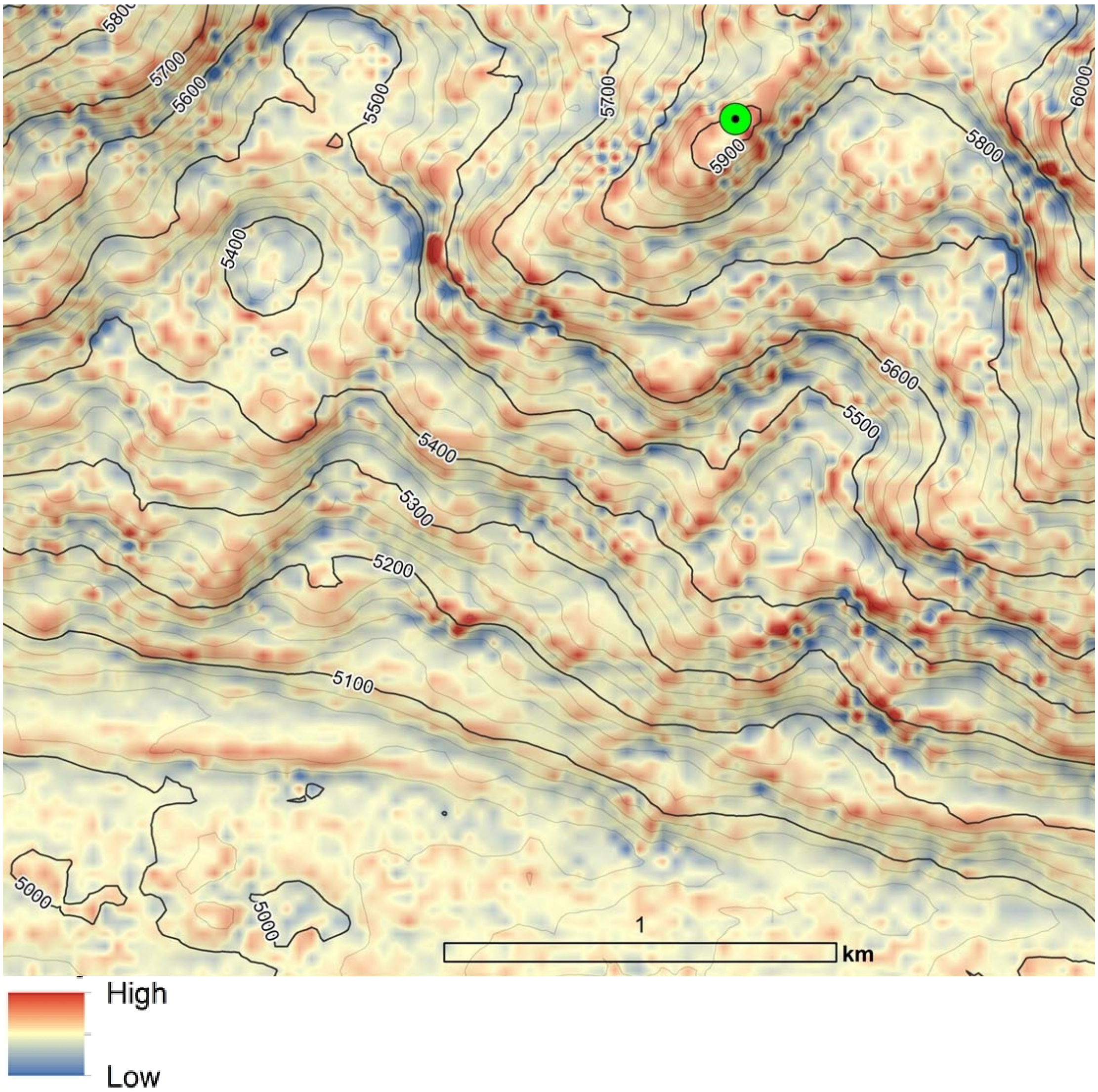

Figure S5: An example of the landscape scale surface curvature as determined using Arc-GIS using merged profile curvature and plan curvature. The slope governs the overall rate of movement downwards. Aspect defines the direction of flow. The profile (i.e. horizontal) curvature affects the acceleration and deceleration of flow and, therefore, influences erosion and deposition. The plan (i.e. vertical) curvature influences convergence and divergence of flow. Considering both plan and profile curvature together allows us to understand more accurately the flow of water (snow-meltwater runoff), snow (avalanche) and unidirectional air masses across a surface (http://authors.library.caltech.edu/25021/2/cabook.pdf).

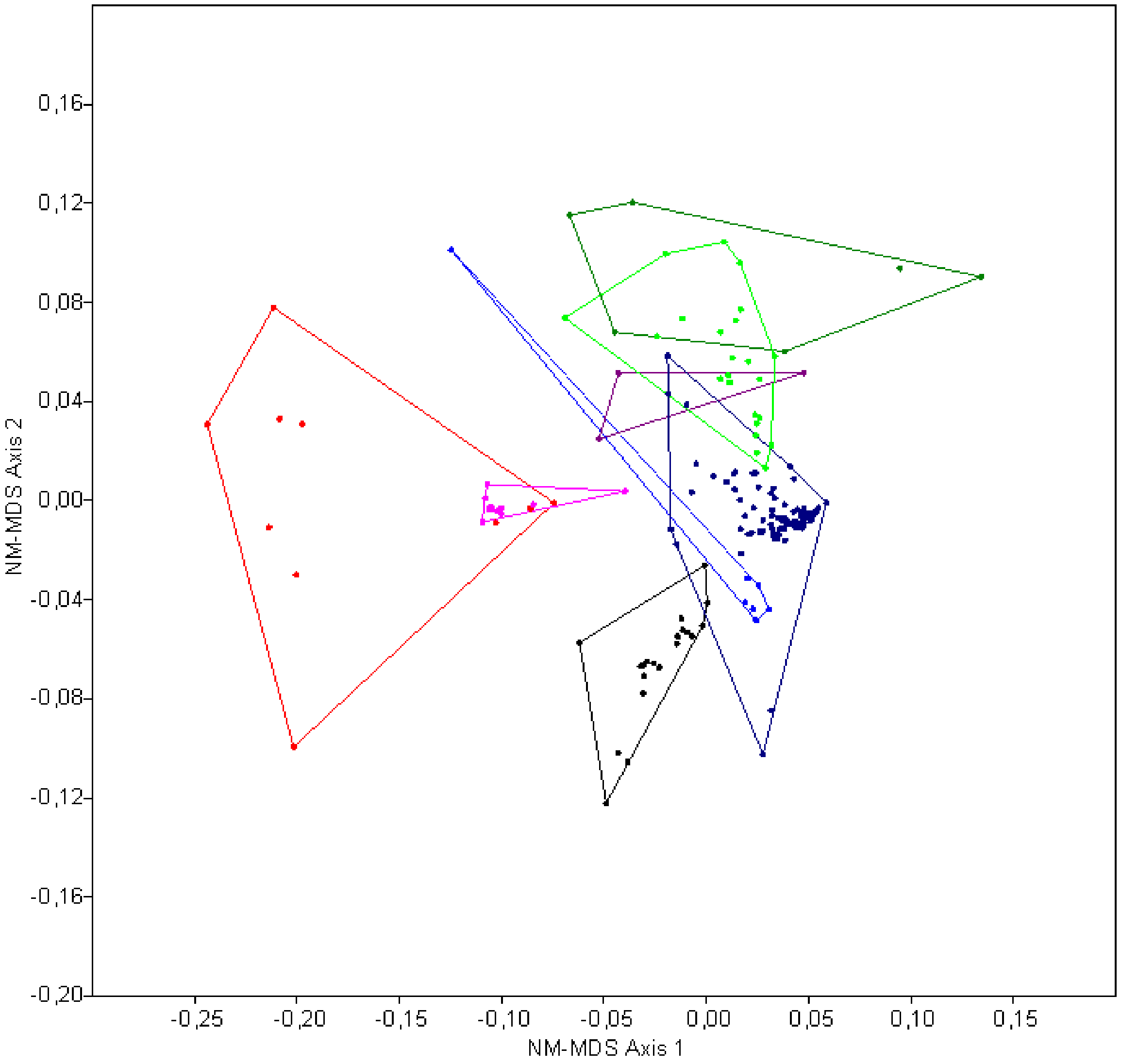

Figure S6: NM-MDS ordination of bacterial microbial communities sequenced from 142 samples of **temperate**, **volcanic**, **Himalayan**, **Antarctic**, **Spitzbergen** permafrost**,** **marine**, **freshwater (lake and river)** and **contaminated** soils and sediments [21].

A)

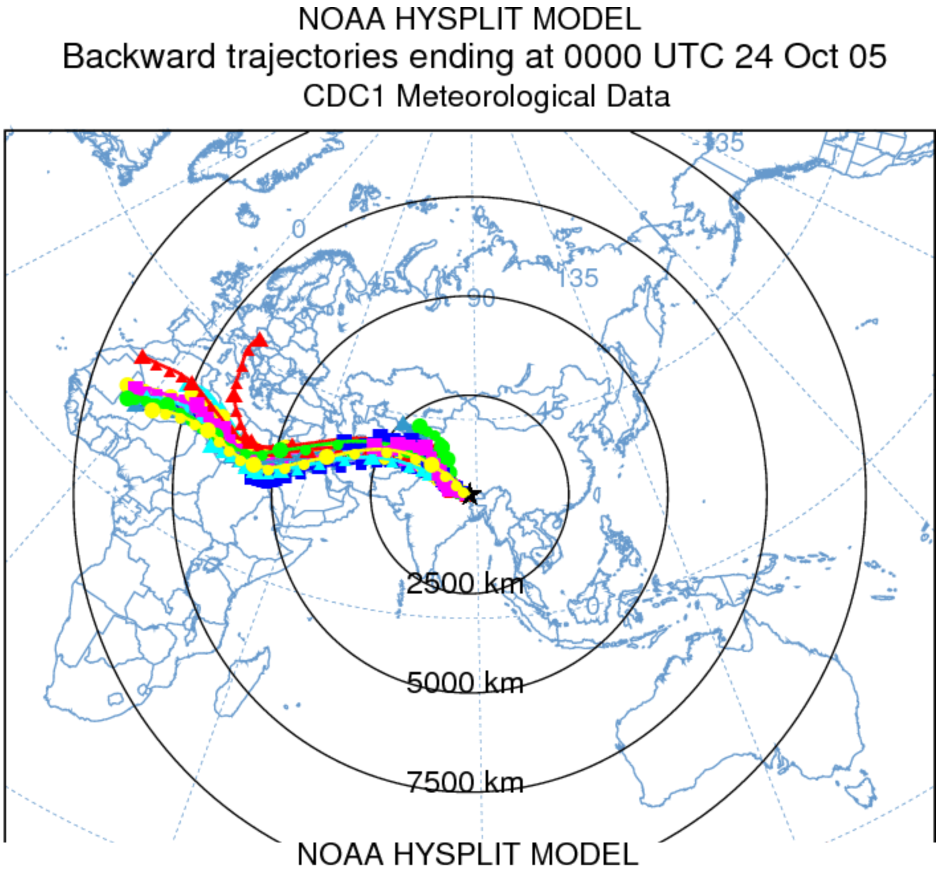

B)

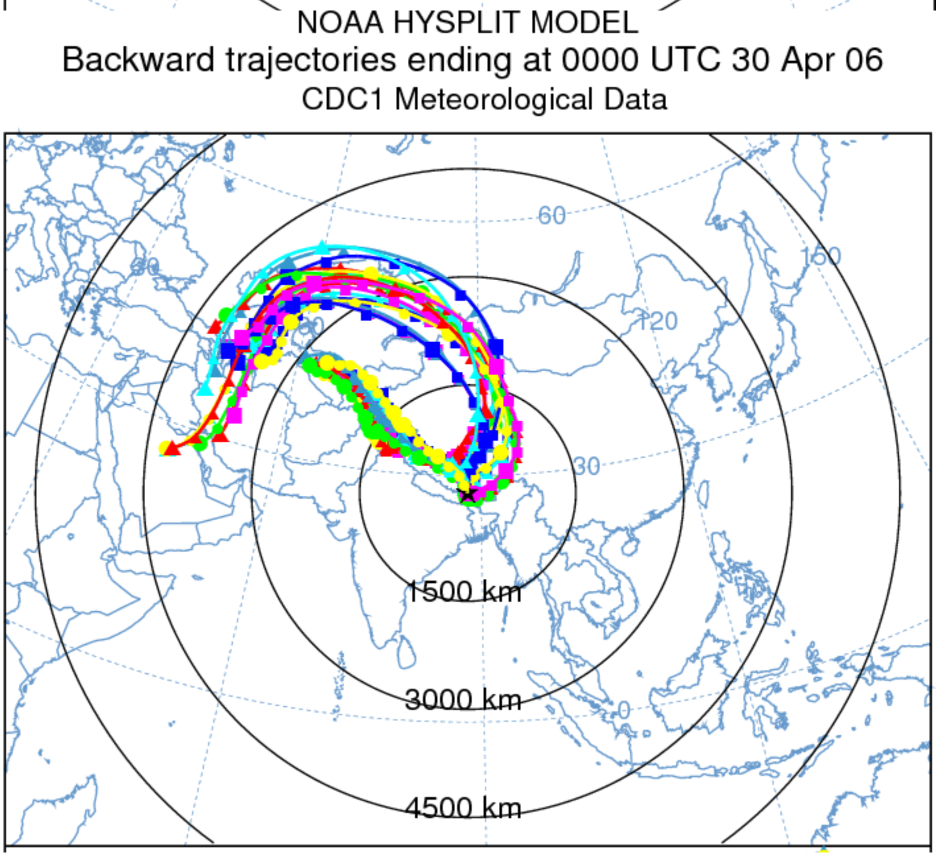

Figure S7: An example of characteristic (A) autumn and (B) spring trajectories of air masses during the dust sampling and snow collection campains. HYSPLIT_4 (Hybrid Single Particle Lagrangian Integrated Trajectory; http://ready.arl.noaa.gov/HYSPLIT.php) Model access via OAA Air Resources Laboratory, Silver Spring, USA (http://ready.arl.noaa.gov) was used to compute back trajectories for all sampling experiments at 00.00 site local time.

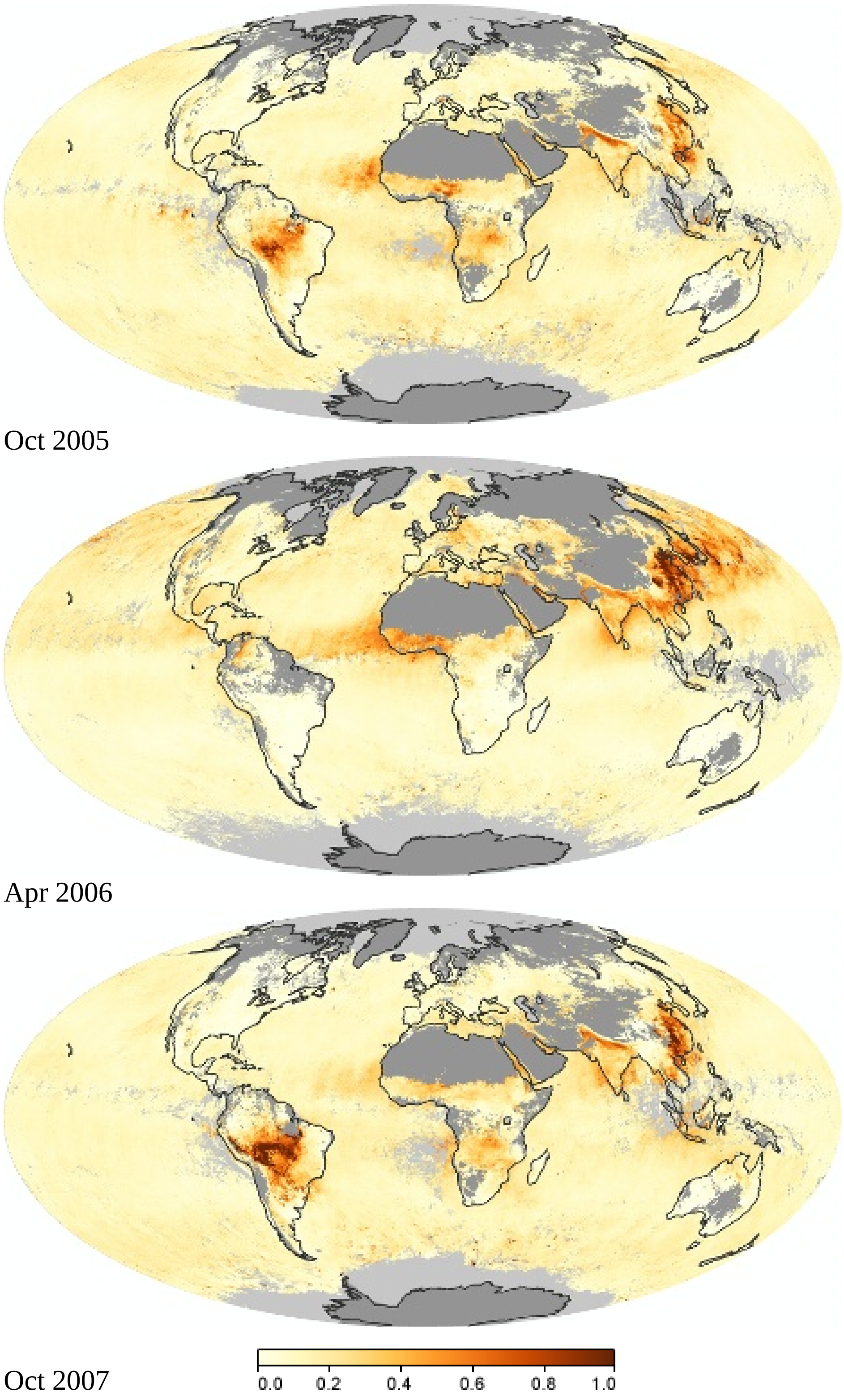

Figure S8: Average monthly aerosol amounts around the world based on observations from the Moderate Resolution Imaging Spectroradiometer (MODIS: http://modis.gsfc.nasa.gov/) on NASA's Terra satellite (http://terra.nasa.gov/). An optical thickness of less than 0.1 (palest yellow) indicates a crystal clear sky with maximum visibility, whereas a value of 1 (reddish brown) indicates very hazy conditions. Source: NASA's Earth Observatory (http://earthobservatory.nasa.gov/).

II. SUPPLEMENTARY TABLES

Table S1: Schematic representation of the sample distribution, sampling seasons and analyses performed in this study: (■/_/_/_) physical-chemical analyses of soil characteristics; (_/■/_/_) pyrosequencing; (_/_/■/_) direct particle and bacterial cell enumeration, size estimation; (_/_/_/■) cultivation experiment at 4°C. Numbers represent number of sampling sites analyzed. See materials and methods for details.

	altitude (m)	2002 october	2005 october	2006 april	2007 october
soil	5000	4/3/_/_			
	5200	4/4/_/_			
	5400	4/4/_/_			
	5600	4/4/_/_			
	5800	4/4/_/_			
	6000	4/4/_/_	4/2/_/_	4/4/_/_	
dust	5000	_/_/3/_			
	5200	_/_/3/_			
	5400	_/_/3/_			
	5600	_/_/3/_			
	5800	_/_/3/_			
	6000	_/_/3/_	_/3/3/_	_/3/3/_	
snow	6000		_/_/7/3	_/_/7/3	_/_/7/3

Table S2: Sequencing coverage of samples from the three sampling periods (Table S1).

2002	Good's coverage
H6000_1	0.74
H6000_2	0.71
H6000_3	0.82
H6000_4	0.85
H5800_1	0.90
H5800_2	0.93
H5800_3	0.91
H5800_4	0.80
H5600_1	0.67
H5600_2	0.85
H5600_3	0.82
H5600_4	0.87
H5400_1	0.56
H5400_2	0.83
H5400_3	0.84
H5400_4	0.89
H5200_1	0.72
H5200_2	0.73
H5200_3	0.80
H5200_4	0.70
H5000_1	0.87
H5000_2	0.95
H5000_4	0.84

2005	Good's coverage
H6000_2	0.81
H6000_3	0.84

2006	Good's coverage
H6000_1	0.80
H6000_2	0.85
H6000_2	0.86
H6000_4	0.43

2005/06 dust	Good's coverage
D6000_2005	0.85
D6000_2006	0.84

Table S3: The percentage distribution of source regions were determined based on the ratio of the number of events of the regions to the total number of sampling events as calculated using HYSPLIT_4 model developed by NOAA/ARL. Back trajectories to source regions were computed for all sampling events on daily basis and expressed as relative distribution.

		Distribution of source regions (%)					
**Distance**	**Direction**	**2002 october**	**2005 october**	**2006 april**	**2007 october**	**Locations**	**Characteristics**
Longrange	W	46	38	61	45	Libya, Egypt, Turkey, Saudi Arabia, Iran, Afganistan, Mediterranean, Indian ocean, Persian Gulf	desert, arid, semi-arid, marine, alpine
Longrange	NW	6	3	10	5	Kyrgizistan, Kazakhstan, Kaspian Sea	arid, semi-arid, lacustrine, alpine
Local (<1000km)	SW	24	30	7	27	Indian subcontinent	aird, semi-arid, alpine
Local (<1000km)	NW	12	3	22	8	Tibetan Plateau	semi-arid, alpine, polar
Local (<1000km)	SE	12	27	0	15	Bay of Bengal, Indian subcontinent	marine, semi-arid
